# Satellite-derived multivariate world-wide lake physical variable timeseries for climate studies

**DOI:** 10.1038/s41597-022-01889-z

**Published:** 2023-01-14

**Authors:** Laura Carrea, Jean-François Crétaux, Xiaohan Liu, Yuhao Wu, Beatriz Calmettes, Claude R. Duguay, Christopher J. Merchant, Nick Selmes, Stefan G. H. Simis, Mark Warren, Hervé Yesou, Dagmar Müller, Dalin Jiang, Owen Embury, Muriel Bergé-Nguyen, Clément Albergel

**Affiliations:** 1 University of Reading, Meteorology Department, Reading, United Kingdom; 2 LEGOS (CNES/CNRS/IRD/UPS), Université de Toulouse, Toulouse, France; 3 Plymouth Marine Laboratory, Plymouth, United Kingdom; 4 Department of Geography and Environmental Management, University of Waterloo, Waterloo, Ontario Canada; 5 H2O Geomatics Inc., Waterloo, Ontario Canada; 6 Collecte Localisation Satellite, Toulouse, France; 7 National Centre for Earth Observation, Reading, United Kingdom; 8 ICUBE-SERTIT, Université de Strasbourg, Strasbourg, France; 9 Brockmann Consult GmbH, Hamburg, Germany; 10 University of Stirling, Stirling, United Kingdom; 11 European Space Agency Climate Office, ECSAT, Harwell Campus, Didcot, United Kingdom

**Keywords:** Environmental health, Limnology, Hydrology

## Abstract

A consistent dataset of lake surface water temperature, ice cover, water-leaving reflectance, water level and extent is presented. The collection constitutes the Lakes Essential Climate Variable (ECV) for inland waters. The data span combined satellite observations from 1992 to 2020 inclusive and quantifies over 2000 relatively large lakes, which represent a small fraction of the number of lakes worldwide but a significant fraction of global freshwater surface. Visible and near-infrared optical imagery, thermal imagery and microwave radar data from satellites have been exploited. All observations are provided in a common grid at 1/120° latitude-longitude resolution, jointly in daily files. The data/algorithms have been validated against *in situ* measurements where possible. Consistency analysis between the variables has guided the development of the joint dataset. It is the most complete collection of consistent satellite observations of the Lakes ECV currently available. Lakes are of significant interest to scientific disciplines such as hydrology, limnology, climatology, biogeochemistry and geodesy. They are a vital resource for freshwater supply, and key sentinels for global environmental change.

## Abstract (summary)

**Table:** 

Measurement(s)	Lake surface water temperature, lake water extent and level, lake ice cover, lake water leaving reflectance
Technology Type(s)	Radar altimeters, radiometers, spectrometers, and multispectral scanner systems
Sample Characteristic - Organism	Inland water
Sample Characteristic - Environment	Satellite imaging
Sample Characteristic - Location	Earth global

## Background & Summary

Lakes hold 87% of liquid surface freshwater on Earth^1^, with the latest census reporting about 117 million lakes covering a small fraction of the Earth’s land surface (3.7%)^2^. Lakes and reservoirs, rivers and wetlands comprise the world’s freshwater ecosystems. The biodiversity they support is a fundamental component of the global biosphere. Lakes provide essential products and ecosystem services and as such they are part of the United Nations’ Sustainable Development Goals dedicated to water resources and to the impacts of climate change^3^. Lakes can be strongly modified by interaction with a changing climate, amplifying external drivers such as the incoming shortwave and longwave radiation, advection and storage of heat within the lake, etc.^4–6^. A substantial body of research demonstrates the sensitivity of lakes to climate^7^ and shows that physical, chemical, and biological lake properties respond rapidly to climate-related changes^8^. Many studies identified the essential lake response variables that act as indicators of the effects of climate change on both the lake and the catchment^8^. Lakes are therefore important carriers of climate-related signals. The Global Climate Observing System (GCOS)^9^ defines “Lakes” as an Essential Climate Variable (ECV) with six linked quantities characterising their physical state, namely, lake surface temperature, water level and extent, ice cover and thickness, and lake water leaving reflectance (colour). All interact and contribute to the lake physical response to climate change. Monitoring these variables closely is a starting point for understanding the complex lake environment and its variations in time and space, a need to which this dataset responds. For convenience, we continue to refer to these variables as “lake” observations also when they are included for water reservoirs, sections of rivers, or lagoons.

Records of lake surface water temperature or lake level spanning about a century (although initially very sporadic) exist for some well-studied lakes such as Tanganyika, Superior, Mendota and Windermere with some observations dated at the start of 20^th^ century^10^. In recent decades, *in situ* and satellite measurements are increasingly used together to quantify lake variability and change, since each observational approach has strengths and limitations. *In situ* data are generally single-point measurements which do not necessarily provide a representative picture of lake responses. For example, intra-lake heterogeneity of thermal response to climate change has recently been demonstrated^11^. Moreover, *in situ* measurements, when available, are collected with different instruments from site to site and rarely measurements are reported with evaluations of uncertainty. However, *in situ* data are indispensable, being direct measurements, including for their use in the validation of remote sensing measurements. Satellite systems offer globally consistent observations including measurements for lakes for which *in situ* measurements are not available. Differing technologies lead to a variety of sampling patterns, resolution, uncertainty and revisit periods. Often, such characteristics have improved upon over the period addressed by the dataset as technology advances. Optical and thermal imagery may be limited by cloud cover, which is not the case for active and passive microwave observations.

The European Space Agency Climate Change Initiative (ESA CCI) is generating multi-decadal satellite-based products to serve the climate modelling and climate user community, including the lakes dataset described herein. The dataset addresses all the thematic variables except lake ice thickenss, namely lake surface water temperature, lake ice cover, lake colour (as lake water-leaving reflectance), lake water level and lake water extent spanning the period 1992–2020. The variables are derived from measurements by instruments including radar altimeters, radiometers, spectrometers, and multispectral scanner systems on satellites such as Sentinel-3, Sentinel-2, Terra, Aqua, Envisat, ERS-2, MetOp, Landsat, Topex/Poseidon, Jason, GFO, Saral/AltiKa and Cryosat. The observations of the five thematic variables derived from data of multiple sensors and satellites consequently span different temporal and spatial resolutions. The observations for each of the thematic variables derived from the different sensors have been regridded to create a multi-variable dataset with a common spatio-temporal representation.

Data are provided on a common regular 1/120° latitude-longitude (about 1 km by 1 km) grid and with daily temporal coverage. The Lakes ECV product consists of data that have been regridded and aggregated (when required) across observations from multiple platforms (level-3 super-collated files, L3S). The daily files contain observations for 2024 inland waterbodies distributed globally and spanning a wide range of ecological settings and characteristics. Each variable is accompanied by a per-datum uncertainty, with the exception of lake water level and extension for which one value is reported for each lake. Lake surface water temperatures are additionally accompanied by per-pixel quality levels, which reflect an assessment of the validity of the datum and its uncertainty estimate. The uncertainty estimate for each thematic variable is at a different stage of maturity, as detailed in the Method section.

The thematic variables have been individually validated against *in situ* measurements (when available, otherwise with other appropriate methods) and the level of consistency among these variables has been explored. Exploring the physical processes occurring in the lakes have highlighted some issues especially in the thematic variables for which the retrieval is less mature and more complex. This particularly benefits the optical retrieval of the lake water leaving reflectance during periods of sub-pixel or thin ice cover, and biogeochemical variables derived from the lake water leaving reflectance (turbidity and the phytoplankton pigment chlorophyll-*a*).

At the time of this study, the dataset presented in this paper is the longest and most complete collection of satellite observations of the Lakes ECV, it is ‘analysis-ready’ and it responds to the GCOS ECV monitoring requirements.

## Methods

The dataset contains data products from multiple thematic variables of the Lakes ECV consistently stored in a regular spatial and temporal grid. The collection of satellite observations includes the following five physical variables which capture specific climate responses of global inland water bodies:

- Lake Water Level (LWL): fundamental for understanding the balance between water inputs and water loss.
- Lake Water Extent (LWE): elucidates lake expansion (e.g. glacial regions) and the effects of drought (e.g. arid environments). It also determines the locally cooling effect of water bodies.
- Lake Surface Water Temperature (LSWT): correlated with regional air temperatures and a proxy for mixing regimes, driving biogeochemical cycling and seasonality.
- Lake Ice Cover (LIC): freeze-up in autumn/winter and break-up in spring are proxies for gradually changing climate patterns and seasonality.
- Lake Water-Leaving Reflectance (LWLR): a direct indicator of biogeochemical processes and habitats in the visible part of the water column (e.g. seasonal phytoplankton biomass fluctuations), and an indicator of the frequency of extreme events (peak terrestrial run-off, changing mixing conditions).

The dataset is the result of four years of the ESA CCI Lakes consortium joint effort but it has roots in methodological development spanning multiple decades. Each of the thematic lake variable datasets has been derived from observations by different instruments and with different retrieval techniques. Therefore, the procedures used in producing the data are described for each variable separately. In particular, input and ancillary data, the core algorithm(s), outputs, uncertainty estimates and quality indicators are detailed as relevant for each thematic ECV in the dataset. A detailed description is provided in the ESA CCI Lakes Algorithm Theoretical Basis Document^12^.

**Fig. 1:**
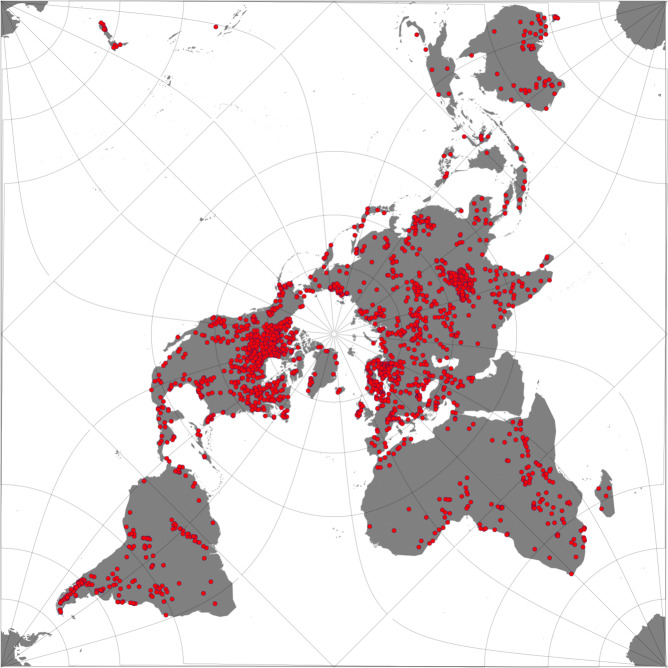
Geographical distribution of the 2024 lakes where the retrieval of the thematic ECVs has been performed.

The spatial coverage of the dataset presented in this paper includes 2024 lakes and reservoirs (Fig. 1), selected to be a globally representative sample of the largest inland waters, which cover a wide range of ecological settings and characteristics. The lakes included in this collection are deemed suitable for remote sensing methods based on a priori expectations of their morphology. The maximum distance to land^13^ has been used to select the target water bodies. Given the variable instrument resolution, the maximum distance to land gives an estimate of how likely a lake (or a portion of it) will be observed and how likely the observed pixels are to (seasonally or occasionally) include land. Since the spatial resolution of the presented dataset is approximately 1 km × 1 km, only lakes with distance to land greater than 1 km were selected, with the exception of ten lakes that were of particular interest for LWLR which is retrieved with higher resolution instruments.

Figure 1 shows the geographical distribution of the lakes. The list of the lake selection is available at the project website https://climate.esa.int/en/projects/lakes/data as a csv file and through a link CEDA which can be accessed through the lake dedicated website at the University of Reading (http://www.laketemp.net/home_CCI). The link is https://gws-access.jasmin.ac.uk/public/cds_c3s_lakes/CCI_LAKES/CCI_LAKE_LIST_v2/LAKE_LIST_MASK_CCI_v2_UoR_fv1.0.html.

### Lake water level

#### LWL input data

The LWL product is derived using the so-called Geophysical Data Records (GDRs) made available publicly by the space agencies (CNES, NASA and ESA). It includes the re-tracking range, the altitude of the satellite, the geographical position of the reflecting surface and the corrections such as geophysical and propagation in the atmosphere. Radar altimetry data from Topex / Poseidon, Jason-1, Jason-2, Jason-3, GFO, ERS2, Envisat, Saral/AltiKa, Cryosat-2, Sentinel-3A and Sentinel-3B, from 1992 until nowadays was used to generate the LWL product. The input data streams are summarised in Table 1, where the time range and the typical local time of observation are reported.

**Table 1:** Characteristics of level-2 data for the instruments used for LWL.

Instrument	Satellite	Dates used (dd/mm/yyyy)	Typical local time of observation
Poseidon-1	Topex/Poseidon	26/09/1992 to 18/01/2006	non-sun-synchronous orbit
Poseidon-2	Jason-1	07/12/2001 to 01/07/2013	non-sun-synchronous orbit
Poseidon-3	Jason-2	20/06/2008 to 10/10/2019	non-sun-synchronous orbit
Poseidon-3B	Jason-3	17/01/2016 to 31/12/2020	non-sun-synchronous orbit
Radar Altimeter (RA-2)	Envisat	01/03/2002 to 08/06/2012	10:00 h
AltiKa	Saral	25/02/2013 to 01/07/2016	10:00 h
SAR Altimeter	Sentinel-3A	16/02/2016 to 31/12/2020	10:00 h
SAR Altimeter	Sentinel-3B	01/01/2019 to 31/12/2020	10:00 h

#### LWL ancillary data

Polygons of lake contours from the Hydrolake database^14^ were used to determine the satellite tracks that cross each of the lake in the database and the measurements that must be considered. A threshold for each lake is also used for filtering outliers: it is based on a priori information on the historical ‘speed’ (or rate) of water level changes. If compared to the previous LWL value, the instantaneous rate of change overpasses the threshold, the LWL is removed from the time series. At the level of individual altimetry measurements, an ‘editing’ is also used based on a threshold on the backscatter of the reflected energy (it must be ranged between 7 and 40 dB). When a lake surface is too smooth, it provokes a so-called sigma bloom with very high backscattering and quasi-specular echo which cannot be processed by the waveform re-tracking algorithm. If the backscatter is below 7 dB, the reflecting surface is not considered as being cover by open water. When several satellite tracks cross a lake, a bias is estimated between each track. It includes altimeter bias^15^ and geoid errors^16^.

#### LWL core algorithm

The principle of radar altimetry is to send an electromagnetic pulse towards the nadir of the satellite and to measure the time between the emission of the pulse and its echo on the illuminated surface. Multiplied by the speed of light, it gives the distance *R* (called range) between the satellite and the reflecting surface. On-board an ultra-stable oscillator is used to measure the propagation time of the pulse within the atmosphere.

The ellipsoidal height *H* of the reflecting surface is given by the following relation:

1 $$H=a-\left(R+\sum {C}_{p}+\sum {C}_{g}\right)+B$$

where *a* is the altitude of the satellite above the ellipsoid of reference. The ellipsoid height is then converted to the altitude *h* by considering the local undulation of the geoid *N*:

2 $$h=H-N$$

The terms *C*_*p*_ and *C*_*g*_ are corrections essentially of two types: the propagation corrections (*C*_*p*_) is related to the fact that the radar pulse propagates through the atmosphere at a speed lower than the speed of light and the geophysical corrections (*C*_*g*_) is related to vertical movements of the Earth’s surface (e.g. solid earth and pole tides) for which we want to correct the measurement to a fixed reference in the Earth’s reference frame. Loading effect due to ocean tide are not considered. *B* is the bias which has been calculated in case tracks of several satellites are used over a given lake. When only one track crosses the lake, as is usually the case for small lakes, bias estimation is not required. When each individual value of *H* has been calculated, outliers’ values are removed using a 3-sigma filter, and then all *H*’s values are averaged in order to calculate a LWL product per pass. A final filtering is then applied on the full LWL time series, using the a priori threshold on the rate of change of LWL. More details on altimetry data processing for LWL are available^17^.

#### LWL output of core algorithm

LWL is calculated for each pass with the associated uncertainty estimated as the standard deviation of the distribution of each individual measurement of *h* along the track over the lake.

#### LWL uncertainty estimate

It is practically impossible to establish a generic error budget for LWL calculation using satellite altimetry because the sources of errors are numerous and they vary strongly from one lake to another, and between altimeters. However, for large lakes, whichever satellite mission is considered, and in normal lake surface conditions (no specular echoes) the main source of uncertainty comes from the wet tropospheric correction and, depending on the regions, it varies from 1 to 5 cm. The wet tropospheric correction, which is precisely measured from onboard radiometers, is only estimated using climate models since the footprint of radiometer on the ground covers several hundreds of km^2^, which makes them inappropriate for lakes. Therefore, the correction from climate models is more appropriate. The combination of altimeter noise and geophysical corrections may then be as high as 8–10 cm in the worst cases, but it usually remains at 3 to 5 cm. For small and narrow lakes, the uncertainty on LWL actually varies between 10 cm and 1 m (above which the data is discarded). In such cases, the uncertainty depends on the shape of the echoes (waveform) and the ability of the re-tracking to interpret it analytically in terms of the range between the satellite and the lake surface. Under very poor conditions (very narrow lakes for example) the re-tracking used (OCOG^18^) may be not robust enough to retrieve the range without large uncertainty. In such cases, another factor influencing the final uncertainty is the altimeter itself: with Saral/AltiKa and with Sentinel-3A/B SAR altimeters, the impact of lake morphology on the result is reduced since the footprint is also drastically reduced compared to former missions, as Topex/Poseidon or Jason-1, with Low Resolution Mode (LRM) altimeters. It has been shown^19^ that with Saral/AltiKa the accuracy increases by a factor 2 to 5 (based on comparison to *in situ* LWL in Chile and Argentina). It is, moreover, worth to note that with the current altimeters, based on SAR measurements, the range of uncertainty (checked using sets of *in situ* data on small lakes) has been significantly improved by at least a factor 2^19,20^. It is now not unusual to obtaine sub-decimeter accuracy for lakes of few km^2^ or for narrow reservoirs, which was nearly impossible to obtain with LRM altimeters. More details on LWL uncertainty estimation are given in the LWL validation section.

#### Quality indicators and data gaps

The quality of the LWL estimate is given by the dispersion indicated by the standard deviation of the level-2 measurements along the transect over the lake for a given day. Past and present missions, a constellation of nadir altimeters, do not cover the 2024 target lakes in the database. Moreover, different families of satellites (a set of satellites with the same orbit, as in the case of the Jason’s or ENVISAT/ERS missions) are in different orbits and cover a different set of lakes. As a consequence, some lakes are partially covered in time, generating data gaps for some lakes over the whole 1992–2020 period of the dataset. Consequently, for many lakes, there are also data gaps with time series covering only a portion of the whole 1992–2020 period of the dataset.

### Lake water extent

#### LWE input data

Considering the strategy adopted to calculate LWE (see below), for each lake we collect a set of optical satellite images spread out over whole period of time when LWL was measured from satellite altimetry. This, in general, covers the period from 1992 to 2020 for large lakes. In other cases where only recent altimeter data were available for the LWL calculation (for example from the Sentinel-3 constellation), it covers only the period from 2016 to 2020.

We have used Landsat-5 Thematic Mapper (TM), Landsat-7 Enhanced Thematic Mapper-plus (ETM+) and Landsat-8 Operational Land Imager (OLI), and the Sentinel-2A/B Multi Spectral Instrument (MSI). Landsat images are available on the USGS GLOVIS image archive (http://glovisusgs.gov) and the Sentinel-2 images on the ESA Science Hub (https://scihub.copernicus.eu). The input data streams are summarised in Table 2, where the time range and the typical local time of observation are reported.

**Table 2:** Characteristics of level-2 data for the instruments used for LWE.

Instrument	Satellite	Dates used (dd/mm/yyyy)	Typical local time of observation
MSS	Landsat-4	26/09/1992 to 18/01/2006	09:45 h
TM	Landsat-4	07/12/2001 to 01/07/2013	09:45 h
MSS	Landsat-5	20/06/2008 to 10/10/2019	09:45 h
TM	Landsat-5	20/06/2008 to 10/10/2019	09:45 h
ETM+	Landsat-7	01/03/2002 to 08/06/2012	10:00 h
OLI	Landsat-8	25/02/2013 to 01/07/2016	10:00 h
MSI	Sentinel-1	25/02/2013 to 01/07/2016	18:00 h
SAR	Sentinel-2A	16/02/2016 to 31/12/2020	10:30 h
SAR	Sentinel-2B	16/02/2016 to 31/12/2020	10:30 h
ASAR	Envisat	01/01/2019 to 31/12/2020	10:00 h
SAR	ERS-1	01/01/2019 to 31/12/2020	10:00 h
SAR	ERS-2	01/01/2019 to 31/12/2020	10:00 h

#### LWE auxiliary data

Polygons of lake contours from the Hydrolake^14^ database are used to determine the region of interest which must be considered for each lake. In few cases, these polygons have to be redrawn in order to fit in a better way with the region of interest.

#### LWE core algorithm

To calculate LWE we use a combination of a lake’s water surface extent and water height at different dates, and then establish a relationship between these variables called hypsometry. This calculation has been used in many studies^21–25^. LWL historical time series inferred from satellite altimetry are used to determine when the lake was at low, medium and high level. We, then, collect images covering these different periods. The hypsometry is then adjusted ideally using a set of about 10 to 15 pairs of (*LWL*, *LWE*) and a simple least square adjustment. Knowing the function *LWE* = *f*(*LWL*) we can relate *LWL* from altimetry to *LWE* using the hypsometry equation. This allows us to achieve a high temporal resolution without intense image processing requirements. Depending on the shape of the hypsometry (linear or quadratic) we decide a priori whether the hypsometry will be a first or second order polynomial. In the first case, the hyspometry equation is

3 $$LWE\left(t\right)=a\;LWL\left(t\right)+b$$

while in the second case it is

4 $$LWE(t)=a\,LWL{(t)}^{2}+b\,LWL(t)+c$$

where *a*, *b* and *c* are the coefficient of the hypsometry.

To measure *LWE* which allows to calculate the hypsometry curve, many methods exist to extract water surface from satellite imagery, which can be divided into single-band and multi-bands methods with a thresholding approach as well as with more complex techniques such as neural network algorithm. We use the classical method of water detection based on index combining a multi-band ratio such as the Normalized Difference Water Index (NDWI), the Modified Normalized Difference Water Index (MNDWI), the Automated Water Extraction Index (AWEI) of visible green and near infrared (NIR) or short-wave infrared (SWIR) bands introduced by McFeeters^26^ and also Feyisa^27^. The water mask is then derived from thresholding the index. In addition, another method was used which allows an optimal threshold to be selected by reducing the within-class variance, or by maximizing the between-class variance^28^ (the OTSU approach). Improvements based on edge detection algorithm have also be implemented. This added feature focusses on the calculation of the histogram over regions with strongly defined edges, which is the expected case at the interface between surface water and land. By doing so, the water signature becomes more strongly represented in the histogram resulting in a bimodal distribution and thus allows for a more adaptive threshold.

#### LWE output of core algorithm

Hypsometry coefficients are produced for each lake and are intermediate values essential for the final calculation of LWE.

#### LWE uncertainty estimate

Water recognition is a challenging task depending of many factors (clouds coverage, floating vegetation, suspended material, complex environment, etc.). It is very problematic to determine the accuracy of LWE product, since it is impossible to measure water extent of so many lakes directly from the ground. The accuracy of the LWE estimates is found to be highly dependent on the type of lake and meteorological conditions during the image acquisition. For simple cases where the lake is well filled and close to its maximum extent and the satellite image is acquired with optimal meteorological conditions (e.g. little or no cloud cover), the results obtained by the different approaches are very similar. In the case of shallow water bodies, a large proportion of the reflected signal could originate from the bottom of the lake rather than from the water surface itself, leading to greater differences in LWE estimates between the different procedures. This may also occur in the case when the lake/water body has a high content of suspended material.

Three approaches have been used to validate the method:

- Field survey: boat campaign with GPS mapping on Lake Chad Archipelago Area
- Comparison of LWE derived from HR and VHR imagery
- Validation considering the uncertainty in hypsometry calculated as the root-mean square error (RMSE) of the fitting

**Fig. 2:**
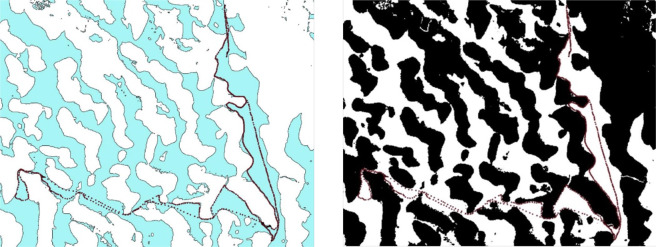
Lake Chad archipelago boat trip (red dots) and classification with simple NDWI threshold method on NDWI (left) and based on OTSU approach (right).

The second and third approach are described in the LWE validation section. Regarding the first approach, in April 2019 a boat campaign has been organized over the Lake Chad archipelago where the lake shore was precisely mapped using a Global Positioning System (GPS) receiver. This region of the Lake Chad presents high contrast of different environments: sandy flat zone together with flooding and non-flooding vegetation and shallow turbid water. However, one may see from Fig. 2 that optical imagery in such case study with both methods (unsupervised OTSU and supervised threshold based on NDWI) capture well the complex water mask measured during the boat trip.

#### LWE quality indicators and/or data gaps

The hypsometries are used only within a limited range of elevation values. Since we used LWL product to derive the LWE, and since we used selected pair of (*LWL*, *LWE*) using water mask inferred from satellite imagery, for all value of LWL which is higher than the highest LWL of the set of pair, or lower than the lowest one, then the LWE is not calculated. It is preferred not to extrapolate the hypsometry relationship since it is a characterization of the bathymetry shape of the lake and may not follow the hypsometry outside of the elevation range used to establish hypsometry. This choice generates many gaps in the time series for extreme values of lake elevation.

### Lake surface water temperature

The algorithms to generate the LSWTs draw on developments in the ESA funded projects ARCLake, the CCI for Sea Surface Temperature as well as UK Natural Environment Research Council project GloboLakes. The main algorithmic steps are: identification of water-only pixels for valid retrieval; inversion of thermal infrared radiances to LSWT based on physical modelling; estimation of total LSWT uncertainty and uncertainty components; evaluation of confidence level for each pixel; cross-sensor LSWT harmonization. The LSWT dataset for this project has been created utilising only daytime imagery and single-view level-1b data.

#### LSWT input data

The input data are level-1b satellite images, which by definition consist of calibrated, geo-located brightness temperatures (BTs) and radiances. The input data streams are summarised in Table 3, where the time range and the typical local time of observation are reported. The ATSRs data include the Along Track Scanning Radiometer (ATSR2) and Advance Along Track Scanning Radiometer (AATSR) v3/v2.1 level-1b archive (http://data.ceda.ac.uk/neodc/aatsr_multimission) while the ATSR1 is excluded for the present. For the dual view instruments, such as the ATSRs and the Sea and Land Surface Temperature Radiometers (SLSTRs) only nadir-view observations are processed.

**Table 3:** Characteristics of level-1b data for the instruments used for LSWT: ATSR2, AATSR, MODIS (Moderate Resolution Imaging Spectroradiometer), AVHRR (Advanced Very-High-Resolution Radiometer), SLSTR.

Instrument	Satellite	Dates used (dd/mm/yyyy)	Typical local time of observation
ATSR2	ERS-2	01/06/1995 to 22/06/2003	10:30 h
AATSR	Envisat	20/05/2002 to 08/04/2012	10:00 h
MODIS	Terra	24/02/2000 to 31/12/2020	10:30 h
AVHRR	MetOpA	01/03/2007 to 31/08/2019	09:30 h
AVHRR	MetOpB	13/12/2012 to 31/08/2019	09:30 h
SLSTR	Sentinel-3A	01/06/2016 to 31/12/2020	10:00 h
SLSTR	Sentinel-3B	21/08/2020 to 31/12/2020	10:00 h

These input data streams share the following characteristics: the sensors observe BTs in channels around 11 and 12 *μ*m at a nadir resolution around 1 km; the platforms are in sun-synchronous orbits with local equator passing times between 09:30 and 10:30 h; in addition to BT observations, there are reflectance channels that are useful in determining that a given BT is obtained while observing only water surface (rather than cloud or a component of land within the field of view). These similarities constrain the properties of lakes that are observable, and help the consistency over time of the LSWT record obtained.

#### LSWT auxiliary data

The auxiliary data mainly give supports to determine if a pixel belong to one of the designated lakes and if it is filled with water, to the radiative transfer model and to the optimal estimation retrieval. In more details:

- A fixed lake mask^29^ is used to determine which pixels are considered for water detection and retrieval. The lake mask is derived from the water bodies mask (v3.0) of the ESA’s Land-Cover (LC) CCI project^30^ and consists of a netCDF file that includes lake identifiers to select the pixels to process and a distance to land to determine if the field of view of a given satellite radiance is wholly or partially on water on the mask, given its centre location and its view angle^13^.
- Numerical weather prediction (NWP) fields are used to linearise and act as a prior to constrain the retrieval. These were acquired from the European Centre for Medium-range Weather Forecasting (ECMWF) re-analysis, generated consistently with a single version of the atmospheric general circulation model and assimilation scheme. The ERA5 data-set^31^ was used for MODIS, ERA-Interim^32^ for the ATSRs and the AVHRRs and the ECMWF operational data stored in level-1b files for the SLSTRs.
- NWP-based values for LSWT (surface temperature) are not currently suitable to provide the prior information for spatial thermal structure in lakes. The LSWT prior therefore is derived from previous generations of the LSWT product, which have been used to generate a spatially complete field of surface temperature climatology using reconstruction strategies.
- Lake emissivity is acquired through a lookup of emissivity derived from the refractive index for a range of wavenumbers, view angles, temperature and wind speed^33,34^.
- Fixed error covariance matrices (uncertainty information) are input parameters to the LSWT retrieval scheme.

#### LSWT core algorithm

The LSWT algorithm is based on a per-pixel processing consisting of four main components: the identification of the lake pixels filled with water, a forward model (the radiative transfer model is used), the LSWT retrieval based on optimal estimation and finally the remapping into a regular grid.

Water detection (WD) is applied to potential inland water pixels that belong to a lake. WD operates by calculating a score against several metrics, derived from the reflectance channels available. For this reason, the LSWT is obtained only during daytime for this version of the dataset.

The WD score for a given metric is defined as a linear ramp between 0 and 1 (with thresholds *t*_0_ and *t*_1_ on the metric), similar to well-known concepts of fuzzy logic (the scores act qualitatively as probabilities). The metrics for scoring are reported in Table 4. The first two metrics use the expectation that reflection from a cloud-affected pixel exceeds that from a clear view of a lake, with values appropriate to different wavelengths. The MNDWI is the Modified Normalised Difference Water Index^35^ and the NDVI is a Normalised Difference Vegetation Index^36^. The setting of the thresholds was done within the NERC GloboLakes project using AATSR imagery tuned to a probability of cloud image derived from the Medium Resolution Imaging Spectrometer (MERIS) 300 m imagery. The tuning of thresholds was done one-at-time across metrics, maximising the posterior probability that a certain pixel is cloudy or cloud free.

**Table 4:** Thresholds test for water detection.

X, metric	Score definition	Thresholds
R_870_	$$s=\left\{\begin{array}{cc}0 & if\;X\ge {t}_{0}\\ \frac{X-{t}_{0}}{{t}_{1}-{t}_{0}} & if\;{t}_{0} < X < {t}_{1}\\ 1 & if\;X\le {t}_{1}\end{array}\right.$$	t_0_ = 0.097, t_1_ = 0.022
R_1600_	$$s=\left\{\begin{array}{cc}0 & if\;X\ge {t}_{0}\\ \frac{X-{t}_{0}}{{t}_{1}-{t}_{0}} & if\;{t}_{0} < X < {t}_{1}\\ 1 & if\;X\le {t}_{1}\end{array}\right.$$	t_0_ = 0.048, t_1_ = 0.012
MNDWI	$$s=\left\{\begin{array}{cc}0 & if\;X\le {t}_{0}\\ \frac{X-{t}_{0}}{{t}_{1}-{t}_{0}} & if\;{t}_{0} < X < {t}_{1}\\ 1 & if\;X\ge {t}_{1}\end{array}\right.$$	t_0_ = 0.295, t_1_ = 0.515
NDVI	$$s=\left\{\begin{array}{cc}0 & if\;X\ge {t}_{0}\\ \frac{X-{t}_{0}}{{t}_{1}-{t}_{0}} & if\;{t}_{0} < X < {t}_{1}\\ 1 & if\;X\le {t}_{1}\end{array}\right.$$	t_0_ = −0.085, t_1_ = −0.245
MNDWI-NDVI	$$s=\left\{\begin{array}{cc}0 & if\;X\le {t}_{0}\\ \frac{X-{t}_{0}}{{t}_{1}-{t}_{0}} & if\;{t}_{0} < X < {t}_{1}\\ 1 & if\;X\ge {t}_{1}\end{array}\right.$$	t_0_ = 0.375, t_1_ = 0.685

The retrieval scheme is optimal estimation (OE)^37^. OE updates a prior LSWT in the light of the difference between the observed BTs and the BTs expected given the prior LSWT, as evaluated with the forward model. The retrieved state $\widehat{x}$ is derived using the following equation^38,39^

5 $$\widehat{x}={x}_{a}+G\left(y-F({x}_{a})\right)\quad {\rm{w}}{\rm{i}}{\rm{t}}{\rm{h}}\quad G={\left({K}^{T}{S}_{\varepsilon }^{-1}K+{S}_{a}^{-1}\right)}^{-1}{K}^{T}{S}_{\varepsilon }^{-1}$$

stating that the retrieved state $\widehat{x}$ is the prior state plus an increment of *G*(*y*-*F*(*x*_*a*_)). *F* is the forward model and we use the radiative transfer model RTTOV^40^ (version 12.3 for MODIS and version 11.3 for the other sensors) run for the prior re-analysis data and prior LSWT. The matrix *K* expresses how the observations change for departures from the prior state *x*_*a*_, i.e., it is a matrix where a given row contains the partial derivatives of the BT in a particular channel with respect to each element of the state vector in turn. The partial derivatives are the tangent linear outputs from the forward model *F*. *S*_*ε*_ is the error covariance of the differences between the model and observed BTs. This error covariance matrix is the sum of the radiometric error covariance in the observations (*S*_*o*_) and estimated error covariance of the forward model (*S*_*m*_). *S*_*a*_ is the error covariance matrix for the prior state variables. Standard OE theory also enables estimation of the retrieval uncertainty, *χ*^2^ a diagnostic of the retrieval fit and *k* the sensitivity of the retrieval to the true LSWT (*averaging kernel* in retrieval theory). The latter two outputs are used within quality level attribution (see the LSWT quality indicators section).

#### LSWT output of core algorithm

The output of the OE is level-2 (L2) data in swath projection. The remapping from the L2 data to the fixed level-3 (L3) grid at (1/120)° is a destination-pull algorithm where, given the L3 cell coordinate of the centre, the closest L2 pixels LSWT value is assigned to the L3 cell of the L3U (uncollated) file. The polar orbiting satellite carrying the sensors used for this dataset typically complete 14–15 orbits each day resulting in the same number of L2 or L3U files. The LSWT outputs are collated to produce one file for each 24-hour period, corresponding to day-time observations. Following the GHRSST conventions^41^, the selection of the best observation is done choosing the input cells with the highest quality level and if multiple observations have the same quality level, then the average is computed. Finally, an adjustment due to different sensors has been carried out using as reference the LSWT from AVHRR on MetOpA since the validation in GloboLakes indicated a better agreement throughout the lakes with the *in situ* data. The inter-sensor adjustment has been calculated per lake averaging per month and spatially per lake and it has been applied only if enough observations where available to estimate the adjustment for the lake (more than 3 months of data) and if the uncertainty of the adjustment was <0.049 – conditions met for about 800 lakes. For MODIS an adjustment of 0.19 K and 0.11 K has been applied for LSWTs of quality level 4 and 5 respectively for all the lakes. The uncertainty of the bias correction is included in the total uncertainty. For lakes where the bias correction has not been applied the impact of changes in sensor on the long-term trends in LSWT is less well constrained, and trends should be treated with caution.

#### LSWT uncertainty estimate

The standard uncertainty is defined as the standard deviation of the estimated error distribution. Standard uncertainty is evaluated for each pixel at L2 and then propagated into the gridded dataset accounting for the correlation structure of errors between pixels. The overall uncertainty attributed accounts for instrument, retrieval and sampling effects. The instrument part addresses the propagation of error in the satellite observations (the brightness temperatures, BTs) through the retrieval process, using equations appropriate for the type of retrieval used (optimal estimation, see below). The retrieval uncertainty expresses the range of possible LSWTs compatible with the observations even if they were error free, since the intervening atmosphere produces some ambiguity in the relationship between the surface LSWT and the top of atmosphere satellite BTs. Another component of retrieval uncertainty is the influence of the prior value used in the optimal estimate, and error in which also (only slightly) affects the result. The retrieval uncertainty component is also expressed using equations derivable for optimal estimation. Sampling uncertainty at level-3 (gridded data) arises when only part of the lake within the grid cell is observable. This is well parameterised as a function of the fraction and properties of the grid cell that is observable. A limitation of the uncertainty evaluation (and to a lesser extent the retrieval) is use of error covariance parameters within the optimal estimator that are relatively poorly known (this is quite common in optimal estimation approaches). An aspect of uncertainty that has not being accounted for in the retrieval process and not included in the quoted LSWT uncertainty are impacts from any residual cloud or land influences on observations after cloud screening and water detection.

#### LSWT quality indicators and data gaps

The quality indicator for the LSWT is a confidence level from 0 to 5 where 5 indicates the highest confidence. The quality level is a concept distinct from uncertainty: a highly uncertain LSWT can have the highest quality level if all the assumptions needed to derive a valid LSWT and to evaluate its uncertainty are met: the quality level reflects the degree of confidence in the validity of the uncertainty estimate and not the magnitude of data uncertainty. For example, the quality levels are influenced by vicinity of the lake shore where geolocation uncertainty implies a higher probability of mixed land-water pixels. The LSWT uncertainty is valid in areas fully filled with water. The quality level assigned to a pixel will be the lowest level (row of Table 5) that matches any of the conditions shown in the table. The assignments are compatible with GHRSST conventions^41^ where a particular level is given if none of the conditions higher up any column of the table are met. We recommend using quality levels 4 and 5 for climate applications. Some quality level 2 or 3 data may be useful, but cannot be assumed to be useful without detailed inspection. Quality level 1 data are not suitable for use (bad data). For the observations from the MODIS instrument only, quality level 4 and 5 observations have been populated.

**Table 5:** Quality levels definition, where *s*_shore_ is the threshold for the water detection score at a distance 0.5 ≤ *d* ≤ 1.5 (km) and *s*_water_ is the threshold for the water detection score at a distance *d* > 1.5 km to the closest land, *k* is the sensitivity to true LSWT variations, which implies minimal dependence on the prior LSWT information. The LSWT prior and χ2 account for the goodness-of-fit of posteriori simulated and observed brightness temperatures.

QL	meaning	*s*_shore_	*s*_water_	*χ*^2^	*k*	*ϕ*
1	bad data	<0.5		>3	<0.5	
2	worst	<2	<0.5	>2	<0.9	>55
3	low	<3.5	<2	>1	<0.95	
4	acceptable	<4.5	<3.5	>0.35		
5	best		<4.5			

The ESA CCI LSWT data contains gaps in space and time due to clouds and satellite revisiting time. Periods with no observations tend to be longer for smaller lakes and for observations by instruments with a smaller swath width that revisit a given location less frequently. LSWT values are also absent when the lake is considered to be covered in ice.

### Lake ice cover

#### LIC input data

The input data for the LIC product were MODIS (Terra and Aqua) level-1b calibrated and geolocated at-aperture radiances (MOD/MYD02QKM 250 m, bands 1-2; MOD/MYD02HKM 500 m, bands: 3–7 Top of Atmosphere Reflectance), Collection 6.1, obtained from the National Aeronautics and Space Administration (NASA) for the period 2000–2020. The input data streams are summarised in Table 6, where the time range and the typical local time of observation are reported.

**Table 6:** Characteristics of level-1b data for the instruments used for LIC: MODIS.

Instrument	Satellite	Dates used (dd/mm/yyyy)	Typical local time of observation
MODIS	Terra	24/02/2000 to 31/12/2020	10:30 h
MODIS	Aqua	04/07/2002 to 31/12/2020	13:30 h

#### LIC auxiliary data

Three auxiliary datasets are used as part of the LIC product generation. The first dataset provides a raster delineation of lakes based on the maximum water extent derived from the land/water mask available from ESA’s CCI Land Cover (v4.0) at 150 m resolution.

Two other datasets are used in a preprocessing step, prior to entering the main processing chain, to flag which of the lakes are likely to form or not to form ice. This step is introduced to narrow down the number of lakes to process globally as a mean to decrease computational costs. The datasets used for this step consist of ERA5 ice depth (thickness) data provided by the ECMWF^31^ and ice thickness determined from Canadian Lake Ice Model (CLIMo^42^) simulations. Ice cover is deemed possible to form on a lake if ice depth is determined to have reached a thickness greater than 0.001 m on any day from either CLIMo or ERA5 over the period 2000–2020. In the case of discrepancies between ERA5 and CLIMo derived ice cover, and for alpine regions where deep glacial lakes are found (e.g. European Alps, Southern Alps of New Zealand, and the Andes), an additional check is done for these lakes through visual examination of MODIS RGB reflectance images to determine if any of the lakes form ice over the period of interest (2000–2020). Only lakes flagged to form ice enter the main processing chain for LIC product generation.

#### LIC core algorithm

The LIC processing chain includes three modules: data import, retrieval, and data export. Data is processed one day at a time. As part of global initialization, the water mask is loaded. Then, the data for each day is processed. One execution of the processing chain processes one day of data.

Six MODIS Top of Atmosphere (TOA) reflectance bands and solar zenith angle (SZA) band are used for feature retrieval (i. e. for labelling as water, ice, or cloud)^43^. The reflectance bands are MOD02QKM at 250 m resolution (band 1: 0.645 *μ*m and band 2: 0.858 *μ*m) and MOD02HKM at 500 m resolution (band 3: 0.469 *μ*m; band 4: 0.555 *μ*m band 5: 1.240 *μ* *μ*m for Aqua; band 6: 1.640 *μ*m for Terra; band 7: 2.130 *μ*m). The geolocation is provided at 1 km resolution and is interpolated to 250 m. Prior to applying retrieval, pixels of interest are identified as “good” or “bad” using quality bands from the original MODIS TOA reflectance product.

Pixels of interest are classified and labelled as cloud, ice or water from a random forest (RF) algorithm^43^. As an ensemble approach, RF integrates decision trees developed by bagging samples to improve the limitations of the single-tree structure^44^. The bagging creates several subsets randomly from training samples with replacement (i.e. a sample can be collected several times in the same subset whereas other samples are probably not selected in this subset). Subsequently, each data subset is used to train a decision tree. For building a single tree, a random sample with a few variables is chosen as split candidates from all variables. The number of variables available to a split is one of the key RF hyperparameters. For the whole RF model, the number of trees is defined a priori to develop various independent classifier outputs. The final class of each unknown sample is assigned by the majority vote of all outputs from the trees.

Labelled pixels are resampled to a 1/120° grid output grid each day. Aggregation from individual swaths is performed by taking a majority vote between ice and water, ties broken by selecting water. If there are zero ice and water pixels, then the cell is labelled as cloud if there are non-zero cloud pixels; otherwise the output cell is labelled as “bad”.

#### LIC output of core algorithm

The output of the LIC processing chain consists of daily files mapped onto a 1/120° grid. Each file contains three bands: Band 1 (lakes flagged as 1. not forming ice and 2. forming ice); Band 2 (Class labels: 1. water, 2. ice, 3. cloud, and 4. bad); and Band 3 (uncertainty reported as % classification error: 0.83 for water, 2.23 for ice, and 3.07 for cloud).

#### LIC uncertainty estimate

The assessment of uncertainty in the LIC product is currently performed through computation of a confusion matrix built on independent statistical validation. Thus, uncertainties are not assessed at a pixel level, but rather from classification error calculated from multiple samples/images. The reference data for validation are collected from the visual interpretation of imagery from a large number of lakes globally and over several ice seasons (freeze-up and break-up periods) by skilled ice analysts. Classification error (expressed as %) derived from the confusion matrix is the metric used to report total uncertainty for each class (ice, water, cloud). Pixels belonging to the same class are simply assigned the same % error value in the uncertainty band provided. Probability of class belonging, which informs about confidence in the classification, is considered as a valuable proxy for the uncertainty associated with machine learning (ML) algorithms. It is not currently provided in the ESA CCI LIC product v2.0.2 but planned in future releases.

### Lake water leaving reflectance

#### LWLR input data

The primary input data for LWLR products were level-1b data from the MERIS sensor on Envisat (3^rd^ reprocessing) and the Ocean and Land Colour Instrument (OLCI) sensor on Sentinel-3A and Sentinel-3B. The data were obtained from ESA for the period 2002–2012 and 2016–2020, respectively. MODIS level-1b data were also acquired from the National Aeronautics and Space Administration (NASA) for the period 2009–2019, to provide overlap with and continuity between MERIS and OLCI observation periods. The input data streams are summarised in Table 7, where the time range and the typical local time of observation are reported.

**Table 7:** Characteristics of level-1b data for the instruments used for LWLR: MERIS, MODIS and OLCI.

Instrument	Satellite	Dates used (dd/mm/yyyy)	Typical local time of observation
MERIS	Envisat	29/04/2002 to 07/04/2012	10:00 h
MODIS	Aqua	08/04/2012 to 24/04/2016	13:30 h
OLCI	Sentinel-3A/B	25/04/2016 to 31/12/2020	10:00 h

#### LWLR auxiliary data

The delineation of water bodies for LWLR was based on a set of polygons describing their maximum water extent at a resolution of 150 m, based on the land/water mask of the ESA CCI Land Cover project v4.0^45^, manually corrected to exclude upstream or downstream rivers or dams. The polygon data are available in well-known text format^46^ and are identical to those included in the Copernicus Land Monitoring Service (https://land.copernicus.eu) where the selection of lakes overlaps.

#### LWLR core algorithm

The LWLR processing chain was *Calimnos* v1.4 developed at Plymouth Marine Laboratory with specific contributions from the University of Stirling (NERC GloboLakes and ESA CCI Lakes projects) and Brockmann Consult (Copernicus Land Monitoring Service and ESA CCI Lakes projects). In brief, level-1b MERIS data were first geometrically (using AMORGOS) and radiometrically (SNAP v.7.0) corrected. MERIS, OLCI and MODIS pixels identified as water by Idepix (v7.0) were subjected to atmospheric correction with POLYMER v4.13, to provide the LWLR product.

The optical diversity and complexity of inland water bodies can lead to ambiguous interpretation of LWLR in terms of lake biogeochemical properties. Algorithms to derive substance concentrations from LWLR are therefore selected and applied only within a predefined and validated scope. Their assignment is based on a set of 13 lake Optical Water Types (OWT)^47^ following fuzzy classification^48^. The OWT class similarity metric is the spectral angle^49^, highlighting similarities in the shape of the LWLR spectrum with the established OWTs. An additional two non-water reflectance signatures, equivalent to OWTs but derived from near shore observations in small lakes, were added to identify the effect of adjacent land on observations over water.

The applicability to global lake observations, corresponding to a given subset of OWTs, of each of the algorithms selected for chlorophyll-a (Chla) and turbidity was established using *in situ* observation data from LIMNADES (Lake Bio-optical Measurements and Matchup Data for Remote Sensing: https://limnades.stir.ac.uk) to tune specific algorithm coefficients, to define their applicable concentration range, and to characterize their respective uncertainty. This meta-dataset combined data from 25 sources from over 200 inland water bodies across the globe. The tuning techniques described by Neil *et al*.^50^ were used to assign Chla and turbidity algorithms to specific OWTs, as shown in Tables 8, 9, respectively. The same set of Chla and turbidity algorithms are used with both MERIS and OLCI products based on *in situ* data corresponding to MERIS observations, whereas MODIS algorithms were independently optimized.

**Table 8:** Chlorophyll-a algorithms per sensor and Optical Water Type.

Sensor	Optical Water Type	Algorithm reference
MERIS / OLCI	3, 9, 10, 13	OC2 from https://oceancolor.gsfc.nasa.gov/atbd/chlor_a
	2, 8, 11, 12	708/665 empirical band ratio based on Gilerson *et al*.^90^
	1, 4, 5, 6	Semi-analytical NIR-Red band algorithm for MERIS based on Gons *et al*.^91^
	7	Adapted QAA algorithm according to Mishra *et al*.^92^
MODIS	1, 5, 7, 9, 12, 13	OC2M from https://oceancolor.gsfc.nasa.gov/atbd/chlor_a
	2, 3, 8	OC3M from https://oceancolor.gsfc.nasa.gov/atbd/chlor_a
	4, 6, 11	748/667 empirical band ratio based on Dall’Olmo *et al*.^93^
	10	OC2-HI from https://oceancolor.gsfc.nasa.gov/atbd/chlor_a

**Table 9:** Turbidity algorithms per sensor and Optical Water Type.

Sensor	Optical Water Type	Algorithm reference
MERIS / OLCI	1, 7, 10	Based on Zhang *et al*.^94^
	2, 4, 6, 8, 12	Based on Vantreportte *et al*.^95^
	3, 5, 9, 11, 13	Based on Binding *et al*.^96^
MODIS	1, 12	Based on Miller and McKee^97^
	2, 6, 11, 13	Based on Ondrusek *et al*.^98^
	3, 5, 9	Based on Chen *et al*.^99^
	4, 8, 10	Based on Petus *et al*.^100^
	7	Based on Zhang *et al*.^101^

For each pixel, the algorithm results corresponding to the three OWTs with the highest similarity scores were averaged using the OWT membership score as weighting factor to get the per-pixel weighted blend of each biogeochemical product. This procedure reduces discontinuities in Chla and turbidity maps when selected algorithms are applied near the edge of their applicable range.

#### LWLR output of core algorithm

Outputs of the core LWLR processing chain include the unprojected, atmospherically corrected LWLR for all input satellite wavebands of each sensor, the OWT scores, each biogeochemical algorithm and their OWT-based weighted-blended products and the pixel identification flags. For each LWLR band and the blended Chla and turbidity products, uncertainty estimates are also included (see below). The published LWLR product is then the result of reprojection to the global 1-km grid over daily aggregation periods (using averaging in case of multiple observations on the same day), and masking values where the influence of land is evident or where extreme values are encountered.

#### LWLR uncertainty estimate

The LWLR uncertainty model was developed by comparing *in situ* and satellite observations. The propagation of the uncertainty model to individual satellite observations (pixels) is done for each waveband as well as for the derived biogeochemical variables. The validation procedure for LWLR yields uncertainty models per sensor-waveband combination expressed as the relative uncertainty (RU, %) and relative unbiased uncertainty (RUU, %). The latter uncertainty product ignores systematic bias and reflects the uncertainty in LWLR which can be propagated into the Chla and turbidity products (the systematic bias is already removed by individually optimizing the algorithms). The application boundary constraints of the LWLR uncertainty models were determined based on the range of observations in the matchup dataset from LIMNADES. If an uncertainty model could not be established (e.g. *in situ* data were too sparse) or if the LWLR amplitude is out of the application range, the observation is given with unknown uncertainty.

The uncertainty models corresponding to specific biogeochemical algorithm-OWT combinations for Chla and turbidity were expressed as a function of OWT class membership score. Each model was evaluated against the full match-up dataset available for the observation period of the satellite sensor. This is done to ensure that the uncertainty model captures the reduction of uncertainty with increasing OWT class membership, which is ultimately how the per-pixel uncertainty is generated. A weighted-blending of the uncertainty for the three OWTs with the highest similarity scores was then applied to obtain the final uncertainty of each pixel, using the same weighting as for the blended biogeochemical product^51^.

#### LWLR quality indicators and/or data gaps

The associated uncertainty product for each LWLR variable can be used as an approximate indicator of product quality, and it is further noted that the uncertainty product is expressed in absolute percentage values^51^. The LWLR product is the result of calibrated at-sensor radiance following removal of atmospheric effects and contaminated pixels over water area, and therefore it contains gaps spatially and temporally resulting from cloud cover, ice cover, land adjacency, sun glint, white cap, satellite coverage and satellite revisiting time, etc. The full time-series of the LWLR product is a combination of satellite data from MERIS, MODIS, and OLCI, for the period of Apr 29^th^ 2002 to Apr 7^th^ 2012, Apr 8^th^ 2012 to Apr 24^th^ 2016, and Apr 25^th^ 2016 to Dec 31^th^ 2020, respectively. The MERIS and OLCI LWLR data are provided for all of the 2024 lakes included in this dataset. In contrast, a subset of 38 lakes time series include MODIS data to fill the gap between MERIS and OLCI in the current product version, from approximately 250 evaluated for inter-sensor stability and other time-series remaining to be investigated for a future product version.

## Data Records

The ESA CCI Lakes dataset^52^ represents a consistent and homogeneous data record generated from very different sensors. The version presented here is v2.0.2, it can be downloaded at the Centre for Environmental Data Analysis (CEDA) archive (10.5285/a07deacaffb8453e93d57ee214676304) and it is organised in global daily files in NetCDF4 classic format (Network Command Data Form) using the CF (Climate and Forecast) metadata convention^53^ (v1.8) and ESA CCI Data Standards (v2.3). The dataset title, full name, description, and data volume are given for ESA CCI Lakes products in the Table 10.

**Table 10:** Data record information for the gridded (level-3S), multi-sensor, multi-variable ESA CCI Lake product.

Dataset title	ESA CCI Lakes Version 2.0.2
**Full name**	European Space Agency Lakes Climate Change Initiative: Lake products, V2.0.2
**Basic description (to quote in citation)**	Daily-mean lake variables (lake water level, extent, surface water temperature, ice cover, water leaving reflectance) presented on a 0.0833° (1/120°) lat/lon grid, with gaps between available daily observations, and spanning 1992 to 2020 for 2024 lakes distributed globally
**Total data volume**	519.7 GB
**Total number of files**	10353

Not all the thematic ECV products are offered for all the 2024 lakes and throughout the full period. All lakes are covered by LSWT, LWLR and LIC, but not LWL and LWE due to the more limited along-track spatial coverage of radar altimeters. The temporal coverage for each is reported in Table 11. In the version v2.0.2 of the dataset, each of the variables is present in the files only within the period indicated in the table.

**Table 11:** Covering time for each of the thematic ECV product.

Thematic ECV	Start	End
LWL	26/09/1992	31/12/2020
LWE	26/09/1992	31/12/2020
LSWT	01/06/1995	31/12/2020
LIC	24/02/2000	31/12/2020
LWLR	29/04/2002	31/12/2020

The files contain the variables that define the dimensions of longitude, latitude and time. The time variable is also encoded in the filename. The filename follows the CCI data standards v2.3 and it has the form

ESACCI-LAKES-<Processing Level>-<Data Type>-<Product String>-<Indicative Date>-fv<version>.nc where <Processing Level> is L3S for the dataset presented the processing level and indicates super-collated data where observations from multiple instruments and observation times are combined into a common spatio-temporal grid; <Data Type> is LK_PRODUCTS indicating that the file contains lake products; <Product String> is MERGED to indicate that the product come from more than one platform and sensor; <Indicative Date> is the date of observation and it is encoded as YYYYMMDD; <version>: the version of the dataset. All data are released under the licence Creative Commons Attribution 4.0 International (CC-BY 4.0, https://creativecommons.org/licenses/by/4.0/).

## Technical Validation

All the thematic ECVs of the dataset have been validated against *in situ* measurements, where possible, according to the CCI Product Validation Plan^54^. Validation of the dataset has been carried out through direct comparison between remote sensing products and *in situ* data and/or other remote sensing datasets for each of thematic variable individually and/or through manual inspection of a selection of lakes. The level of spatio-temporal consistency between thematic variables was investigated separately to ensure the data are mutually compatible.

### Lake water level validation

Validation of LWL is performed using sets of *in situ* measurements in different regions. The accuracy of LWL calculated using satellite altimeter is strongly dependent on several conditions: the shape, the size, and the surrounding environment of the lakes. It also depends on the quality of the on-board instruments that have strongly evolved from the radar altimeters launched in the 1990s.

The validation of LWL is essential since the accuracy of LWL calculated using satellite altimetry may differ from one type of lake to another one. Therefore, a large set of *in situ* water level data was collected to cover most of the different cases (small/large lakes, with or without ice cover in winter, mountain lakes, etc.).

The validation of this variable therefore has to be carried out for lakes with different characteristics.

Due to the fact that generally the geodetic reference system used by satellite and *in situ* measurements are not the same, and not tied, the validation consists in most of the cases in inter-comparison of the water level changes after removing some biases between the time series as reported in a large number of papers during the last 20 to 25 years. For example, already with the very first measurements of Topex/Poseidon launched in 1992, Morris and Gill^55^ have evaluated the accuracy of altimetry over the Great Lakes in North America, being sub-decimeter compared to the *in situ*. Global inter-comparisons between *in situ* worldwide and altimeter over several missions have also been performed. Using the OCOG retracking algorithm, the accuracy ranges between few centimeters for large lakes to few decimeters for small lakes^56,57^. In many studies, dedicated analysis has been done for specific mission in order to assess the accuracy of measured LWL: for example on Sentinel-3 it has been shown that the SAR mode strongly improves the accuracy once specific algorithm of waveform retracking is used^20,58–60^. Some other studies have been conducted on the assessment of performances of Jason-2^61^, Cryosat-2^62^ or Saral/AltiKa^63^. Other studies focused on the validation of satellite altimetry over specific lakes^15,58,64–68^ or on group of lakes in specific environment^19,55,59,69^.

If in such analysis the *in situ* data are considered as ground truth, there are still difficulties to directly compared them since the physical properties and the scaling of the measurements are different. Satellite measurements are collected along the track of the satellite with a footprint of several square kilometres, while the *in situ* measurements are generally taken on the lake shore very often far from the satellite track by several kilometres. Moreover, the reference system of each of the measurements are different, and the uncertainty on the geoid over the lake adds a source of bias between both types of measurement. In addition, technical issues with gauges, data gaps, human error in collecting the data can easily increase the gauge error level to few centimeters, making them an unreliable source of ground truth for small variations of lake level.

The inter-comparison between the two types of dataset is also complex because the *in situ* measurements are generally daily or monthly average values, while the frequency of the satellite flight over the lake is fully determined by its orbit. The comparison requires then an interpolation, which can also be a source of error, especially when the lake water level variations are abrupt, or in presence of seiche for example.

The errors of satellite altimetry over lakes depend on several factors. Depending on the size and shape of lake shore, the altimetry telemetry waveform which is analysed for the calculation of water level over the footprint can be more complex than the usual shape of such signal from the open surface over the oceans, or over very large lakes. Footprint over narrow reservoirs, for example, covers in-homogeneous surface over the lake with non-water surface such as vegetation, bare soil, sandbanks, ice. This explain (see Table 12) why the performances of the altimeter over large set of lakes can vary from few centimeters to few decimetres. Moreover, the evolution of the altimetry technique from the Low-Resolution-Mode altimeters on the Jason/OSTM series to the SAR altimetry used with the Sentinel-3 series generates an improvement of accuracy from the oldest time series to the newest ones. For example, over the Issykkul and the Illmen lakes we have obtained a gain of a factor 2 with Sentinel-3 data. For lake Argentino, while the root mean square error (RMSE) over the period 1992–2019 is 16 cm, it falls to 8 cm with a correlation of 0.99 when only Sentinel-3A is used.

**Table 12:** Comparison between *in situ* and altimetry on lakes worldwide (Europe, Africa, South and North America and Asia).

Lake	Country	Mean area (km^2^)	Period	RMSE (cm)
Argentino	Argentina	1466	1992–2019	16
Athabasca	Canada	7900	1992–2018	30
Ayame	Ivory Coast	95	2018–2020	13
Aydarkul	Uzbekistan	3000	2002–2010	12
Aylmer	Canada	808	2008–2013	26
Baikal	Russia	31500	1993–2019	11
Baker	Canada	1780	1992–1994	22
Bodensee	Switzerland	539	2016–2020	18
Bratsk	Russia	5478	1993–2019	30
Caribou	Canada	5650	2002–2017	45
Cedar	Canada	1320	1992–2019	27
Chad	Chad/Niger/Nigeria/Camerun	1540	1992–2008	28
Des Bois	Canada	4350	1992–2019	26
Dubawnt	Canada	3630	2009–2018	18
Erie	USA/Canada	25800	1992–2019	7
Garda	Italy	370	2016–2020	8
General-Carrera	Chile/Argentina	20000	2002–2010	22
Geneva	Switzerland	580	2016–2020	4
Guri	Venezuela	4250	2002–2010	82
Huron	USA/Canada	59570	1992–2019	5
Illmen	Russia	980	1996–2019	35
Issykkul	Kyrgyzstan	6236	2016–2020	1.5
Khanka	China	4400	2000–2010	13
Krasnoyarsk	Russia	2000	2003–2008	37
Kubensk	Russia	407	2016–2019	27
Kumsk	Russia	986	2009–2019	12
Kuybyshevsk	Russia	5900	1993–2019	29
Lacha	Russia	300	2009–2019	20
Ladoga	Russia	18135	1993–2019	7
Luzern	Switzerland	110	2016–2020	8
Manitoba	Canada	4610	2000–2019	14
Michigan	USA	58000	1992–2011	11
Neuchatel	Switzerland	210	2016–2020	6.4
Oahe	USA	1500	2002–2010	45
Onega	Russia	18200	1993–2019	14
Ontario	USA/Canada	19009	1992–2019	10
Saint Jean	Canada	1053	1992–2013	44
Segozersk	Russia	815	2009–2019	12
Superieur	USA/Canada	82200	1992–2019	3
Syamozero	Russia	270	2016–2019	10
Tana	Ethiopia	3600	1992–2006	17
Titicaca	Peru	8372	2000–2005	7
Tsimlyansk	Russia	2702	1993–2019	43
Ust-Ilimsk	Russia	1703	2016–2019	15
Verkhnee-Kuyto	Russia	200	2009–2019	16
Vodlozero	Russia	322	2019	8
Volta	Ghana	8502	1999–2010	53
Williston	Canada	1779	1992–2019	82
Winnipeg	Canada	23750	1992–2019	13
Winnipegosis	Canada	5150	2002–2019	23
Zürich	Switzerland	65	2016–2020	7.5

**Fig. 3:**
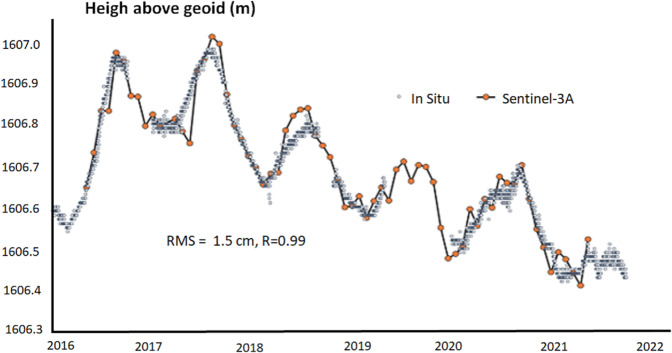
LWL from Sentinel-3A and *in situ* data over the Lake Issykkul (Kyrgyzstan).

We have compared the altimetry product with the *in situ* measurements for 51 lakes spread over all the continents. The results are reported in Table 12. For 11 among the large lakes the accuracy is below 10 cm, for 16 of them it is between 10 and 20 cm, for 14 of them are between 20 and 40 cm and for 7 lakes the RMSE was higher than 40 cm. The comparison for Lake Issykkul in Kyrgyzstan is reported in Fig. 3.

### Lake water extent validation

Validation of water extent is challenging since *in situ* measurements generally do not exist^25,70^. Field work for surveying the water bodies is limited to walking along the shore with a GPS tracking, or using a boat or kayak to follow the shore but it remains limited to very small number of lakes^71^. It is indeed not always possible to walk around lake, since many lakes are not easily accessible. Moreover, in case of shallow water, the exact distance between the boat and the shore, for example 10 m (or more) is equivalent to 2 to up to10 Sentinel pixels. Water extent extraction from satellite observations generates a large amount of uncertainty and once inter-comparisons between radar or optical imageries using different types of methods are done, selection of images to construct the hypsometry curve can be done based on statistical analysis of the dispersion of the coupled variable: LWL/LWE. An additional way to evaluate the accuracy of the LWE extraction is to compare the lake contour from satellite sensor used with high resolution (HR) and very high resolution (VHR) imageries like those provided by the Pléiades constellation^72^.

Another problem is the difficulty to precisely define the real contour of a lake, since a lake can be surrounded by wetlands or covered by floating vegetation. In addition, the definition of lake could include the ice cover other than the open free water area.

Many tests have been done before we could produce LWE for the CCI dataset.

In order to select the appropriate methodology for optical imagery used for the LWE production, we have implemented an inter-comparison process with VHR data using the Pleiades constellation^73^. The comparison requested pair of HR and VHR images acquired within a very short time. The data have to cover the targeted lake as a whole, and the surrounding areas. In most of the cases of the CCI lakes that are large lakes, this is not possible. Moreover, the VHR data are not freely available and therefore the approach can be a costly.

We have acquired two pairs of Pléiades HR data, 70 cm of spatial resolution, a panchromatic channel and 4 visible ones from blue to near infrared channels, on the 30^th^ of December 2019, with a delay of one day with Sentinel-2 and on the 6^th^ of January 2020, which is the same date as the Sentinel-2 acquisition used to determine the contour of lakes. Lake de Der and lac d’Orient in East of France were chosen for this comparison. Lake du Der, medium size and dynamic reservoir located within an overlapping part of Sentinel-2 tracks, allowing up to 14 acquisitions by month, was an ideal case to compare the LWE derived from HR Sentinel MSI at 10 m, and the VHR Pleaides imagery at 0.7 m resolution.

When comparing the LWE derived with Pléiades and Sentinel-2 data acquired within 24 hours, the difference in term of surface were very low, i.e. 29.07 km^2^ for Sentinel-2, and 30.58 km^2^ for Pleiades. 95% of Pléiades Water was recognized by Sentinel-2. There is a very low level of commission of 0.05 km^2^.

**Fig. 4:**
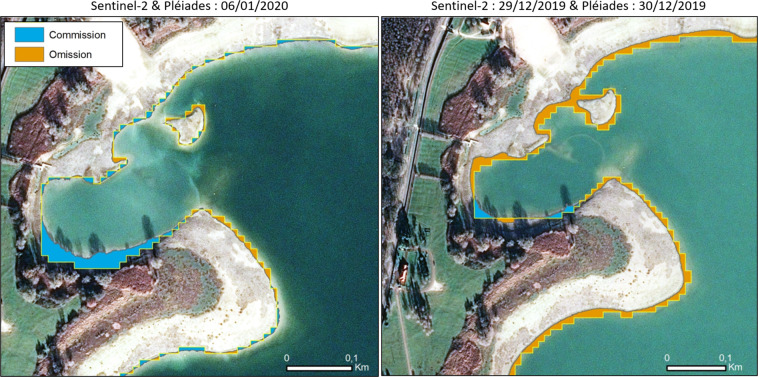
Comparison of LWE accuracy derived with one day of delay between the Sentinel-2 and Pléaides acquisitions and the same day (left).

When the acquisition of the VHR and HR images was the same day, 95.5% of Pléiades water was recognized by Sentinel-2. Of course, there is an effect of resolution i.e. 10 m versus 0.70 m. The shoreline is much finer on the VHR derived LWE. When data are acquired on the same day, the space occupied along the shore of the omission is very narrow, the shoreline corresponds to a staircase of swa-tooth’s effects alternating omission and commission pixel, related to the difference of spatial resolution. Where, the LWE represents two stages of infilling, we observe a large omission belt around the lake shore. This belt corresponds mostly to an increase of the surface of water within one day. This case study allows also evaluating, in the context of infilling reservoir, the part of the 24 h of delay between the two acquisitions. In such case, we observed a large omission belt around the lake shore. This belt in fact corresponds to the increase of the surface of water within one day. So, of course, what is seen as water on the Pleiades image cannot be described as water on the Sentinel-2 image acquired a day before. This is illustrated in Fig. 4.

**Fig. 5:**
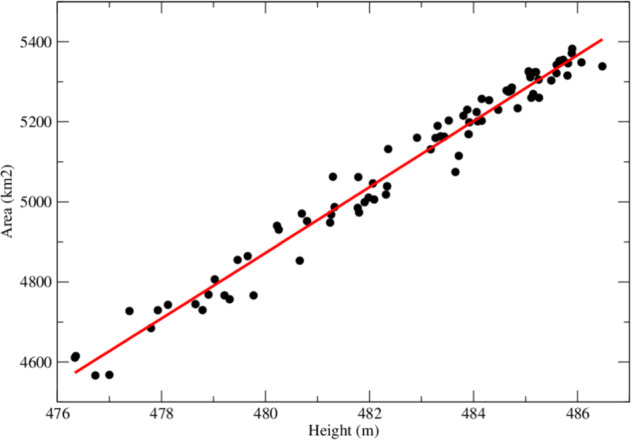
Example of hypsometry precision for Lake Kariba in Africa. The red curve represents the theorical hypsometry determined by least square adjustment of a second order polynomial on the datasets (LWL/LWE). In the case of the lake Kariba, the RMSE is 37 km^2^ which is less than 1% of average extent of 5000 km^2^.

We systematically determine the precision of each time series of LWE calculating the RMSE of the difference between LWE inferred from the polynomial function of the hypsometry of the lake, and LWE directly measured by the satellite image, over the whole dataset of images used to calculate the hypsometry coefficient. Hypsometry has been used only when the RMSE obtained was lower than 10% of the total average extent of the lake. Most of the time, the RMSE kept within 1 to 2%. The idea is also that, when comparing LWE derived with different methodological /sensors approaches the best water times series would produce the best hypsometric curves and therefore statistical correlation can be used to corroborate the method. An example for lake Kariba is reported in Fig. 5.

### Lake surface water temperature validation

The validation of the LSWT is based on matchups between satellite and *in situ* measurements which are collected through personal communication with limnologists and agencies who are willing to share the data for validation purposes.

**Fig. 6:**
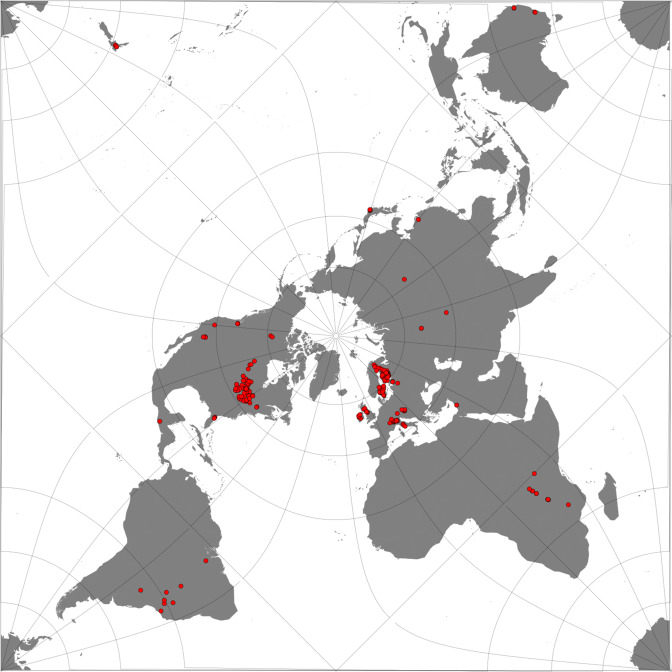
Geographical distribution of the sites of the *in situ* measurements.

The *in situ* temperature data have been collected also through the ARCLake project, the GloboLakes project, the EU Surface Temperature for All Corners of Earth (EUSTACE) project and the Copernicus Climate Change Service (C3S) product. The ESA CCI Lakes LSWT dataset presented in this paper has been validated with *in situ* measurements on 79 lakes with a total of 207 sites. The geographical distribution of the sites is reported in Fig. 6 which shows that most of the sites are situated in North America and Europe, with 38% and 47% of the sites and 24% and 56% of the lakes respectively as shown in Table 13. As the *in situ* data are from a variety of sources, the frequency of the measurements varies greatly between the sources. Only for 90 of the 207 sites measurements with frequency of less than a measurement per day are available, mostly in North America. A portion of the lakes that have been used for the validation are small lakes, for which the LSWT retrieval is most challenging. In Table 13 we have reported the number of lakes for which the maximum distance to land^13^ is less than 3 km. The maximum distance to land^13^ is a meaningful measure of the size of the lakes for LSWT remote sensing. The best resolution of the instruments used for the retrieval of the LSWT is 1 km. If the lake has a maximum distance to land of e.g. 1.7 km such as lake Iseo, the LSWT retrieval is very likely to be available only for that part of the lake and only for a limited proportion of overpasses (clear sky and observations relatively central within the swath). In particular, a combination of factors has to occur: 1) the satellite image locations line up so that some pixels are nominally fully water pixels, which requires the satellite view zenith angle (which affects the on-the-ground resolution) to be such that the half-pixel size is smaller than the distance to shore; 2) these pixels are cloud free; 3) image geolocation errors (which can be of order 1 pixel uncertainty) are small enough that the nominally water-filled pixels are truly water-filled meaning that the water detection tests are passed. Moreover, some of the locations of the *in situ* measurements are situated close to the shore even for large lakes, which means that the nearest water-filled pixels may not overlap the *in situ* measurement, thus increasing the uncertainty in the comparison from spatial representativity.

**Table 13:** Lakes with *in situ* surface water temperature measurements: number of lakes and sites per geographical area, number of small lakes (max distance to land <3 km, number of sites with measurements taken more than once per day up a frequency of 15 minutes.

Geographical area	N lakes	N sites	N small lakes	N sites with HF data
North America	19	79	5	73
South America	5	5	1	4
Europe	44	97	22	8
Africa	3	10	0	0
Central America (Guatemala)	1	1	0	0
Oceania (New Zealand)	2	5	0	4
Japan	1	5	0	0
China	1	1	0	0
Kyrgyzstan	1	1	0	1
Russia	1	1	0	0
Israel	1	2	0	0

The satellite-to-*in situ*-matches are created at the original satellite coordinates, at L2 spatially within 3 km and temporally within 3 hours for the *in situ* data where the measurement time was available. The LSWT of the L3S ESA CCI Lakes dataset are then directly validated to assess the products as seen by users. The validation of the LSWT is performed using conventional and robust statistics of the satellite minus *in situ* measurement difference, the latter being less sensitive to outliers and more descriptive of the majority of data.

**Fig. 7:**
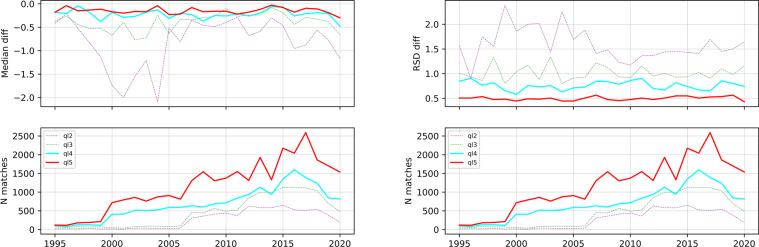
LSWT satellite minus *in situ* temperature difference median (on the left hand side) and robust standard deviation (on the right hand side) per year (upper plot) and number of matches (lower plot) per quality level.

The matchup is carried out per sensor over the 207 locations on 79 lakes. The total number of matches is 81,436 for any quality level and 66,407 for quality levels 2 and above which are reported here. The number of matches varies per year and since the AVHRR and the MODIS sensors have a larger swath than the ATSR sensors (ATSRs swath is 500 km, AVHRRs swath is ~2900 km and MODIS swath is 2330 km), after the year 2000 the number of matches increases as it is shown in Fig. 7a.

In Table 14 the number of matches per quality levels are listed together with the median and the robust standard deviation of the satellite minus *in situ* temperature difference and the traditional metrics, the mean and the standard deviation. The difference between the median and the mean is less than 0.025 K for quality level 5 and it increases as the quality levels get lower suggesting a higher incidence of cold-biased observations for low quality levels, as expected. The best agreement is for quality levels 4 and 5, which are the levels that reflect a higher degree of confidence in the validity of the satellite estimate and that we recommend to use for lake-climate applications in general, although lower quality level data may used only after verifying their fitness for a given lake for their application. Quality level 1 data should never be used and they are classified as “bad data”. Figure 7a,b shows that for high quality levels the median and the robust standard deviation of the satellite minus *in situ* differences are consistently small throughout the years when different instruments have been adopted and a different number of matches is available. They deteriorate as the quality goes lower. The number of matches for quality level 5 is consistently the highest.

**Table 14:** Robust and traditional statistics of the LSWT satellite minus *in situ* difference.

QL	N	Median	RSD	Mean	SD
5	30080	−0.150	0.504	−0.127	0.936
4	17802	−0.220	0.756	−0.249	1.203
3	11767	−0.300	1.023	−0.413	1.472
2	6758	−0.590	1.460	−0.792	1.811
1	15029	−2.280	3.988	−3.474	4.874

A contribution to the difference on average is the expected skin effect. Infrared radiometers are sensitive to radiation emitted between the air-surface interface and 20 mm below the interface while the *in situ* measurements considered here are taken at a distance up to 1 m from the air-surface interface. During the night, the surface of the water is generally cooler than the subsurface by ~0.2 K^74,75^. However, during the day, if the wind speed is low enough, thermal stratification due to solar heating can contribute to a positive offset to the difference in temperature between the radiometric lake surface and the *in situ* measurement depth (up to 1 m). The positive thermal stratification would be expected to be in the range ≪ 1 K for most observations but occasionally of order a few kelvins. The degree of near-surface stratification to be expected in different lakes depends on fetch, weather conditions (radiative balance and wind speed), the depth of *in situ* measurement, and any local vertical mixing perturbations introduced by the presence of the *in situ* measurement system. While several studies have analysed the air-water interface for the sea surface temperature, only few tackled the air-surface interface for inland water where more heterogeneities and larger wind stress variabilities than ocean waters can be found^76,77^. The aggregate effect of these factors is not currently well quantified and they can be different depending on the lake, although a model to quantify the cool skin effect in lakes in presence of natural convection has been recently proposed^77^. In summary, a geophysical contribution to the satellite minus *in situ* temperature difference is the expected skin effect of −0.2 K, but other positive geophysical offsets are similar in magnitude and difficult to quantify precisely for each lake. Additionally, a contribution to the temperature difference can be found in the variety of sources of the *in situ* measurements:

- different instruments have been used for the measurements and we do not have any information on the instruments used
- part of the measurements are not accompanied by an exact timing and/or by an accurate position which can contribute to the difference since LSWT can quickly varies in time and space.

### Lake ice cover validation

Validation of the LIC product is performed through computation of confusion matrices built on independent statistical validation (i.e. from pixels independent to those used for training of the random forest (RF) algorithm). Groups of pixels (Areas Of Interest or AOI) for open water, ice and cloud are collected from a selection of lakes from visual interpretation of MODIS Terra and Aqua images (i.e. MOD02/MYD02) over both the freeze-up and break-up periods interspersed across a 21-year MODIS record (2000–2020). Both MOD02/MYD02 false (R: band 2, G: band 2, B: band 1) and true (R: band 1, G: band 4, B: band 3) colour composites are used as reference images to manually collect AOIs with assigned labels (water, ice and cloud) to assess the accuracy of the LIC product. Validation is performed on Terra and Aqua derived LIC individually, before merging into the daily Terra/Aqua LIC product and before aggregating into the common ca. 1 km × 1 km grid of the multivariate dataset.

**Fig. 8:**
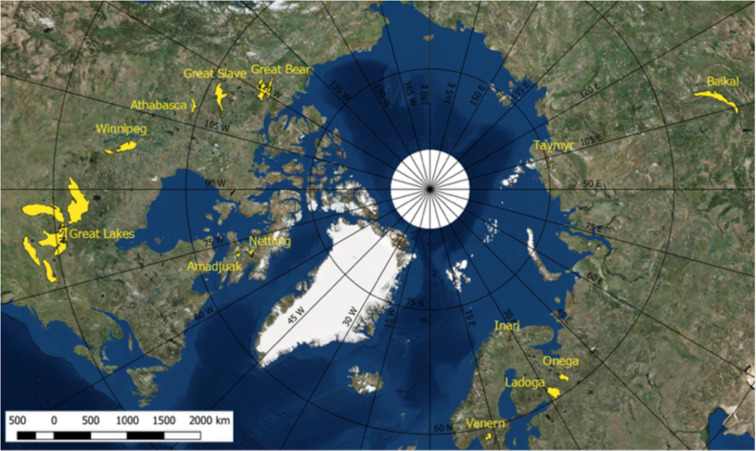
Geographical distribution of lakes used for LIC product validation (Source^43^).

A total of 17 lakes across the Northern Hemisphere currently serves for the purpose of both development and validation of the LIC product (Fig. 8). In a previous assessment of MODIS LIC from Terra only for the same set of lakes^43^, AOIs were collected for three ice seasons (2002–2003, 2009–2010, 2016–2017) as to provide a good temporal spread over the full Terra record to ensure algorithm stability. The RF algorithm provided overall and class-specific accuracies above 98% and a more visually accurate depiction of open water, ice cover and cloud cover than other machine learning algorithms evaluated^43^. The classifier was shown to offer robust spatial transferability over the 17 lakes and to perform consistently well across ice seasons and independently for the freeze-up and break periods.

Here, we further demonstrate the stability of the algorithm over time and between satellite platforms (Terra and Aqua) using Great Slave Lake (Canada) and Lake Ladoga/Lake Onega (Russia) as examples. AOIs were collected from MODIS Terra RGB colour composites for the 2018–2019 ice season. A total of 10,075,081 pixels were selected from 229 swaths over the lakes. For MODIS Aqua, 1,665,188 samples were collected from colour composite images (Great Slave Lake: 156 swaths, Lake Ladoga/Onega: 61 swaths) in 2020, also encompassing a break-up and a freeze-up period. Results presented in Table 15 further support the stability of the RF algorithm for LIC product generation. Accuracies are found to be consistent across classes. RF also produces comparable classification results between MODIS Terra and Aqua. Individual class accuracies are all above 90% which meet uncertainty requirements of 10% set by GCOS for LIC as a thematic variable of Lakes ECV^9^.

**Table 15:** Confusion matrices showing class specific and overall accuracies for LIC product derived from MODIS Terra (top) and Aqua (bottom), individually.

User-defined		Random Forest (classification)			
		Ice	Water	Cloud	Accuracy
LIC MODIS Terra	Ice	1,514,517	4,518	30,057	97.77%
	Water	1,540	1,286,093	9,213	99.17%
	Cloud	199,996	22,265	7,006,882	96.93%
		Overall Accuracy: 97.34%			
LIC MODIS Aqua	Ice	246,032	2,487	4,751	97.14%
	Water	1,743	538,668	4,655	98.83%
	Cloud	19,738	5,324	841,790	97.11%
		Overall Accuracy: 97.68%			

### Lake water-leaving reflectance validation

The validation of LWLR and associated water quality parameters (i.e., Chla and turbidity) is based on match-ups between satellite and *in situ* observations, where the *in situ* data are sourced from LIMNADES. The evaluations were performed for MERIS and MODIS, due to their long operation (2002–2012, and 2002-now, respectively) and coincidence with matching *in situ* data. The number of matchups included in the validation for LWLR, Chla and turbidity ranged from 243 to 2616 for MERIS and MODIS, which were gathered from up to 71 inland waterbodies (Table 16).

**Table 16:** Number of matchups of each variable for each sensor.

Sensor	LWLR	Chla	Turbidity
MERIS	288	2616	660
MODIS	243	949	451

Validation of POLYMER-corrected LWLR for MERIS with *in situ* match-ups was conducted for 11 wavebands from 412 nm to 779 nm, which show significant linear relationships, with highest coefficient of correlation of 0.86 returned in the 560 nm band^51^. As for MODIS, significant linear correlations were also found for all of the 11 evaluated bands (from 412 nm to 748 nm) between POLYMER-corrected LWLR and *in situ* measurements, with the highest R = 0.83 returned at 547 nm^78^.

Note that the validation with *in situ* data for Chla and turbidity was not independent, since the tuning algorithms adopted from the procedure described by Neil *et al*.^50^ to generate Chla and turbidity have already used the same source of *in situ* data. In this context, a validation procedure of a weighted blended Chla product based on the fuzzy OWT classification framework was developed to demonstrate the performance of water quality products^51^. Based on this uncertainty evaluation, it was found that the uncertainty (i.e., ARU, %) of the blended algorithm result shows flat response to the OWT membership scores. This indicates that our proposed procedure successfully removed the uncertainties across different OWTs by blending water quality products from outputs of several pre-assigned algorithms, and therefore making our product globally validated. Although the dataset used for the uncertainty characterization is not fully independent, the validation presented forms a global assessment. As for local performance, the validation results could be better or worse for a single lake, however, this would not alter the optimal configuration of the system nor the global validation.

It is admitted that a lack of *in situ* reference data and a bias towards turbid, productive and large lakes in the datasets do exist, and is somewhat to overcome when the end-to-end validation of Chla and turbidity products derived from LWLR is concerned. Also, a temporal-spatial sampling bias still exists with most of the available *in situ* data having been collected since the launch of MERIS and relatively close to shore. These inherent defects in the *in situ* dataset will add to uncertainties on the validation of LWLR products over a range of water types and lake geophysical and geospatial characteristics.

For a full description of the validation procedure and results the reader can refer to Liu *et al*.^51^ and the ESA CCI Lakes Product Validation Intercomparison Report^78^. A separate follow-on validation of OLCI is still pending considering the limited matchups with *in situ* measurements from LIMNADES due to the relatively short operation period (2016-now), and that the similarities in radiometric performance and waveband configuration with MERIS would allow propagation of the present results to OLCI.

### Consistency of the thematic ECV products in the dataset

The thematic ECVs in the dataset (L3S product) are generated independently with data originating from different satellite sensors. Nevertheless, physical processes link these observations together and create expectations to find some interdependencies and cross-correlations. Moreover, the analysis of consistency between thematic ECVs provides a means for independent quality control of the primary observation data, related to unresolved observation challenges such as sub-pixel ice cover or poor atmospheric transmissivity, recorded differently between sensors and overpass times.

All results presented in this section are based on version 1 of the ESA CCI Lakes dataset. The major differences in version 2, other than wider spatio-temporal coverage, include the reprocessing of LIC with a new machine-learning based retrieval algorithm, which resolves the classification of dried up lake areas as ice. Also, the quality of LWLR products was enhanced by the introduction of two non-water reflectance signatures to classify land adjacency effects. For a full description of analyses and solutions towards consistency, please refer to the Consistency PVIR^79^. Comparisons between LWL and Turbidity (derived from the LWLR product) and between LIC, LSWT and LWLR are reported in this section.

#### Lake water level (LWL) and turbidity (from LWLR)

The LWL product and the turbidity of the water from the LWLR product form a pair of observations with strong cross-correlation.

**Fig. 9:**
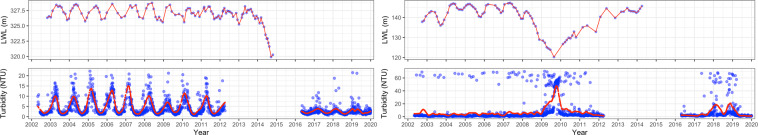
LWL (upper plot) and turbidity (lower plot) time series for the Ilha Solteira in Brazil (on the left) and the Qadisiyah Reservoir in Iraq (on the right). For the turbidity (lower plots), lake median of daily data is shown as blue dots, and the LOESS smoothing is reported as red lines. For LWL (upper plots) the values at the given temporal resolution (blue dots) are connected by the red line.

Figure 9a,b show the daily time series of the Ilha Solteira in Brazil and of the Qadisiyah Reservoir in Iraq respectively where the variations of the two variables are clearly related.

In particular, in Ilha Solteira (Fig. 9a left panels) the seasonal cycle of LWL corresponds with the local precipitation cycle. The rise in LWL corresponds to an increase in turbidity, as expected when more turbid water from land surface run-off (erosion) fills the reservoir. In the example of Qadisiyah Reservoir in Iraq (Fig. 9b right panels), during the years 2007–2009 the outflow of the reservoir was increased to compensate for water level decrease in the river Euphrates, due to prolonged draught^80^. Turbidity (daily median values of all observations for the reservoir) spiked during the minimum LWL. Whereas lower water levels might lead to higher turbidity due to re-suspension of sediments, visual inspection of spatial coverage in the turbidity product also shows an increasing fraction of observations where pixel identified as water may have contained small fractions of land. This has the effect of increasing lake median turbidity estimates. Guided by these results, additional masking of LWLR signatures which suggest an influence of land were added. The turbidity plot for Qadisiyah Reservoir (Fig. 9b right panels) shows a considerable amount of outliers (turbidity higher than 40NTU). These are mainly caused by the land-contaminated pixels.

#### LIC, LSWT and LWLR

The combination of LIC, LSWT and LWLR variables in a single L3S product creates new opportunities to establish the compatibility of individual pixel identification for these products. For example, suspicious cases include ice observations at non-freezing temperatures in the LIC product, and reciprocally, high LWLR where (potentially sub-pixel) ice presence is likely and corroborated by cooling or warming trends in LSWT in adjacent periods.

**Fig. 10:**
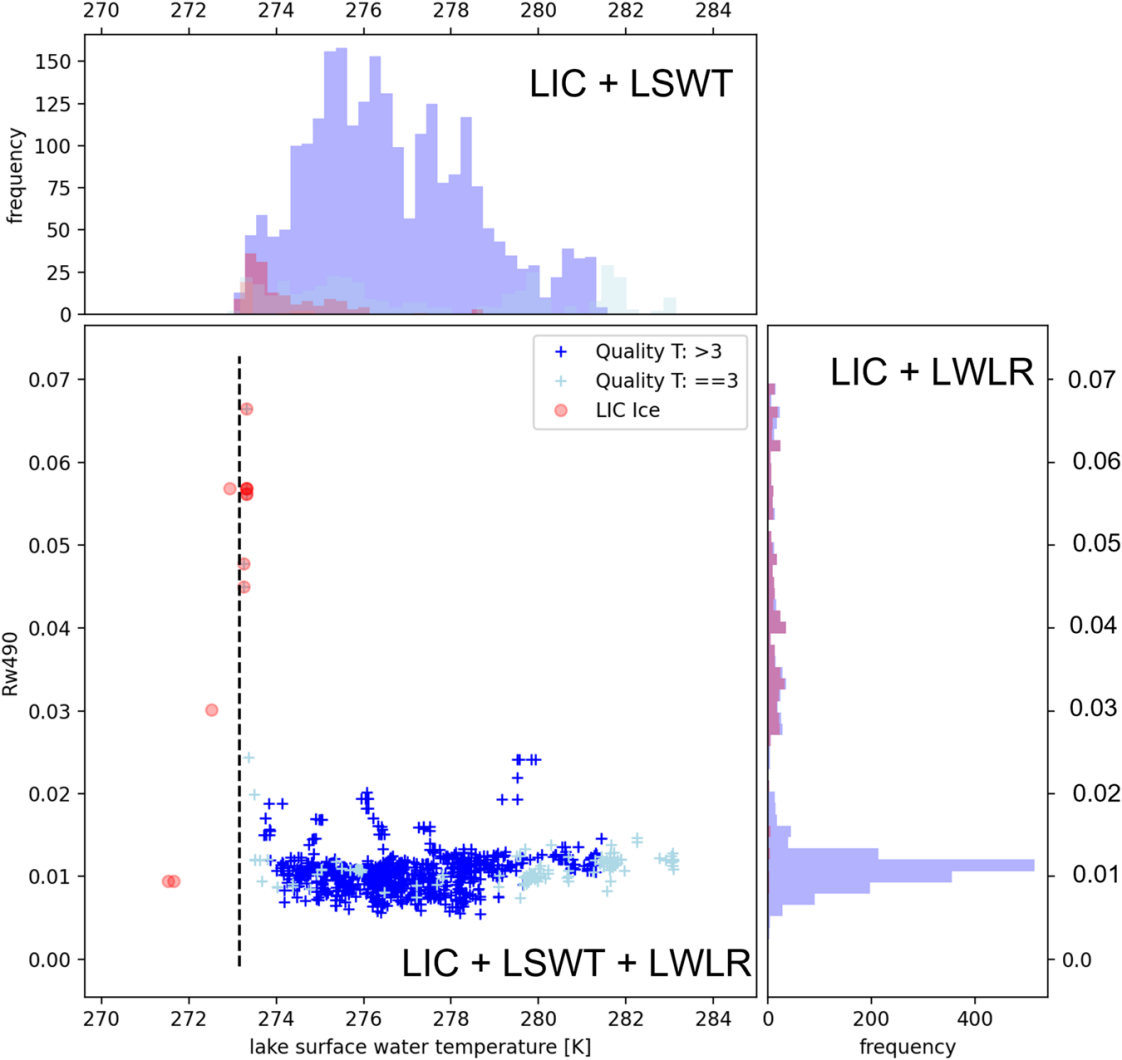
LSWT, LWLR (Rw490) and LIC classification of Lake Amadjuak (Canada) observed on the same day (centre plot). The histograms are based on only two coinciding observations, either LIC/LWLR (right plot), or LIC/LSWT (top plot). If the ixel is classified by the LIC product as ice, it is marked with a red dot. If the LSWT quality is above three (the recommended quality classes) and the pixel is not classified as ice, the data points are marked in dark blue; points of lower LSWT quality (LSWT quality = 3) and not classified as ice are marked in light blue.

In general, the latest version of the thermal products (LSWT, LIC) show a relatively high degree of day-to-day stability, which can be exploited to analyse cases where the influence of observation artefacts is less easily distinguished from noise (e.g. LWLR). To compare the consistency of the LIC classification from MODIS data with the snow/ice flagging of LWLR data from MERIS and OLCI and the LSWT product, we used data extracted at the position of the maximum distance from the shore^13^. This data extract comprises all variables of the L3S ESA CCI Lakes product within a 3 × 3 macro-pixel. Each pixel within the macro-pixel is used as an independent observation. Treating all observations within the macro-pixel independently was considered a reasonable compromise to address data gaps that resulted from sparse data availability between the individual products, caused by using different underlying sensors with varying observation times, and is considered to provide some additional sensitivity to spatial inhomogeneity. Comparisons are shown between lake ice cover classification, LWLR at 490 nm and LSWT in Fig. 10 where macro-pixels from 2002 to 2012 (MERIS) and 2016 to 2019 (OLCI) are considered. Only data points for which observations are obtained on the same day are shown, while there can be several hours between the overpass of individual sensors.

The consistency between the three thematic products is defined as:

- Ice only occurs when temperatures are close to or below freezing point, ignoring variations caused by lake salinity.
- Where ice is observed, LSWT and LWLR cannot be observed.

The identification of ice is the main focus in the LIC product based on MODIS data. All observations of LSWT and LWLR are screened for ice occurrence as well, but with different approaches and based on different sensors and observation times. Where LSWT and LWLR estimates exist, and LIC identifies ice at the same time, LSWT and LWLR products have to be considered suspect. If the temperature is close to the freezing point and the pixel is identified as ice, both LSWT and LIC estimates may still be correct due to different observation times due to melting, freezing, or movement, or due to thresholding for sub-pixel occurrence. On the other hand, if the surface temperature is well above zero degrees and observed with high quality level (4 or 5), the LIC classification can be considered suspicious.

For lake Amudjuak the ice classification of the LIC product appears realistic, as shown in Fig. 10, since the few ice observations all occurred close to the freezing point. Both histograms, which are based on coinciding observations of the two variables, confirm this interpretation. The distribution of LSWT data points which are also classified as ice by the LIC product (red histogram) is tilted towards the freezing point. The data points classified as ice also correspond to relatively high LWLR (histogram on the right, coinciding measurements of LWLR and LIC). These are highly likely observations containing ice, which have not been correctly identified by the pixel identification for MERIS and OLCI data.

For the lake Amadjuak reported here and for other lakes analysed, we found that LSWT and LIC are consistent at a higher degree when compared with LWLR where the influence of observation artefacts can less easily be distinguished from noise. Where LIC and LSWT suggests snow/ice flagging, the LWLR product has to be treated with care and we recommend to avoid their usage. We note that these procedures will be included in future versions (from 2.1) of the LWLR.

## Usage Notes

### ESA CCI Lakes product

Data generated in the ESA CCI Lakes project have been used in five use cases^81–84^, including a case study on Greenland lakes which has been currently submitted to Scientific Report^85^. The case studies were part of the project and they focused on analysing the five thematic variables available in the product: lake water level, lake water extent, lake ice cover, lake surface water temperature and lake water leaving reflectance.

LWL refers to the lake water level above a reference geoid. Radar altimetry from space consists of vertical range measurements between the satellite and water level. Difference between the satellite altitude above a reference surface (usually a conventional ellipsoid and then a geoid), determined through precise orbit computation, and satellite-water surface distance, provides measurements of water level above the reference. Placed onto a repeat orbit, the altimeter satellite overflies a given region at regular time intervals (called the orbital cycle), during which a global coverage of the Earth is performed. Water level measurement by satellite altimetry has been developed and optimized for open oceans. Nevertheless, the technique is now applied to obtain water levels of inland seas, lakes, rivers, floodplains, and wetlands.

LWE can be expressed as the presence of water (on a map), or as the total areal extent of a waterbody (a single number). Studying and monitoring variations and trends in lake area, or lake water extent can be an important tool in identifying climatic variations over time since this physical parameter is regulated by changes in climate. Hence, changes in LWE can be indicators of climate variations since they are sensitive to changes in water and heat balance. LWE together with LWL can be utilized to assess the total volume of water in a lake.

LIC refers to the extent (or area) of a lake covered by ice. Lake-wide ice phenology can be derived from LIC, including freeze onset to complete freeze over (CFO) dates during the freeze-up period, melt onset to water clear of ice (WCI) dates during the break-up period, and ice cover duration derived from number of days between CFO and WCI dates over an ice year^86^). For lakes that do not form a complete ice cover every year or in some years (e.g. Laurentian Great Lakes of North America), maximum ice cover extent (timestamped with date) is also a useful climate indicator that can be determined^87^. Similarly, minimum ice cover extent (timestamped with date) can be derived for High Arctic lakes that do not completely lose their ice cover in summer, although a recent study suggests that these lakes may be transitioning from perennially to seasonally ice-covered^88^. Knowledge of fractional lake-wide ice coverage (expressed in tenth or as a percentage of total area of a lake covered by ice) on a ca. weekly basis is also useful for improving numerical weather forecasting in regions where ice cover forms.

Given the importance of ice cover in lake-atmosphere interactions, the LIC ECV will be of interest to users who wish to: 1) examine short-term trends and inter-annual variability in ice cover globally (ca. 20 years); 2) investigate the impact of changing ice cover conditions on other variables covered in the ESA CCI Lakes project, such as LSWT; 3) conduct data assimilation experiments using state-of-the-art numerical weather prediction systems to demonstrate the impact of better consideration of LIC on, for example, improving predictions of lake-effect snowfall; and 4) evaluate lake models (e.g. FLake) used as lake parametrisation schemes in numerical weather prediction and climate models. Finally, from a socio-economic perspective, the LIC variable may also serve to examine the impact of changing ice conditions on winter transportation (shipping, ice roads) and food security (access to resources by northern communities via ice roads).

LSWT is the surface expression of the thermal structure of lakes and is changing in response to climatic trends. LSWT is needed for climate change studies, water budget analysis (linked to evaporation), lake physical and ecological modelling.

LWLR, also referred to as water colour, is the measurement of the quantity of sunlight reaching the remote detector after interaction with the water column. The maximum depth from which the reflected signal is observed depends on the optical properties of the water column, is dependent on the colour band (waveband) considered and, in natural waters, can range from tens of meters (up to nearly 100 m in the clearest ocean waters) to just centimetres in highly absorbing and/or turbid waters. The colour of water is retrieved using imaging or line-scanning optical detectors on satellites. Each sensor offers a specific trade-off between the observation time (longer periods yielding lower instrument noise) and the spatial resolution as well as the number of discrete wavebands in which reflectance is measured. Because relatively small changes in absorption by, for example, phytoplankton pigment need to be distinguishable, an adequate signal-to-noise of an ocean-colour sensor for the signal received at the top of the atmosphere should be at least 1000:1 (IOCCG 2012).

Based on LWLR estimation, several optical-biogeochemical characteristics of the lake may be determined from its colour. Main quantities of interest are:

- the concentration of phytoplankton pigment, particularly chlorophyll-a, which is found in all species as the major photosynthetic pigment
- vertical transparency, for submerged vegetation habitat mapping or primary production models when combined with chlorophyll-a and temperature observations or models
- the concentration of (coloured) dissolved (organic) matter as a proxy for the dissolved organic carbon pool, as well as the quality of underwater light
- the total amount of suspended sediment (TSM), either expressed as equivalent particulate dry weight or as turbidity.

Currently, globally validated algorithms to retrieve such quantities are available for chlorophyll-a and TSM or turbidity, and vertical transparency, with by far most of the attention in scientific literature dedicated to the retrieval of chlorophyll-a.

#### Reading the products

The ESA CCI Lakes dataset is stored in NetCDF4 Classical format files, compliant to both CF (Climate and Forecast) metadata convention (v1.8) and CCI Data Standards v2.3. The consistency of the product is ensured by the use of a common land mask over a common grid. The main characteristics of the product are as follow:

- The product consists of daily aggregations (the product is specified at the nominal time of 12:00:00 UTC). If a parameter is missing the field is filled with a default value.
- The product provides data over a latitude/longitude grid of 0.0083333° (1/120°), therefore each file contains 21600 number of rows and 43200 number of columns.
- For LWL and LWE, for which the product consists on one value per lake, the value is set on each grid point belonging to a lake according to the common land mask.
- There is a common lake identifier derived from existing databases (Global Land and Wetland Database (GLWD)^89^, Hydrolakes^14^, GloboLakes^13^) and created specifically for the project.
- The extent is −180 to 180 degrees longitude, −90 to 90 degrees latitude, where positive signs point north and east. The pixel coordinate is the centre of the pixel.
- For each variable, the associated uncertainty is also available and for LSWT quality flags are also reported for a correct use use of the product. Quality level 4 and 5 are strongly recommended especially for climate studies.

A wide choice of software packages can be used to visualise or manipulate the NetCDF data. A list of software is provided on the Unidata web site (https://www.unidata.ucar.edu/software/netcdf/software.html). The ESA CCI Lakes files can also be visualised with the Climate Analysis Toolbox (Cate) https://climate.esa.int/en/explore/analyse-climate-data), the reference software for visualising data developed within the CCI Program funded by ESA. Additionally, some scripts allowing to download data only on a selected area or for a selected lake are available in the GitHub of the project (https://github.com/cci-lakes/lakes_cci_tools).

#### Downloading the products

The complete ESA CCI Lakes dataset is available at CEDA 10.5285/a07deacaffb8453e93d57ee214676304 and it is free after registration. It is possible to download the whole dataset (almost 30 years of daily data) or to use scripts in diverse languages (e.g. Python) to download a well defined area in a certain period of time.

#### Will the climate data record be extended in time?

The ESA CCI Lakes dataset described here covers the period from September 1992, when the first altimetry data became available, to the end of 2020. ESA has recently funded a continuation of the CCI Lakes project, which will allow the release of future version of the dataset with new thematic variables such as Lake Ice Thickness (LIT) and Colored Dissolved Organic Matter (CDOM) as well as increased temporal coverage.

#### How should I refer to the products in publications?

The dataset is citable^52^ and by using the data you agree to cite both dataset and this article.

## Data Availability

An option to visualise the dataset is to use the software Climate Analysis Toolbox (Cate) see https://climate.esa.int/en/explore/analyse-climate-data/).
